# Network dynamical stability analysis reveals key “mallostatic” natural variables that erode homeostasis and drive age-related decline of health

**DOI:** 10.1038/s41598-023-49129-7

**Published:** 2023-12-13

**Authors:** Glen Pridham, Andrew D. Rutenberg

**Affiliations:** Department of Physics and Atmospheric Science, Dalhousie University, Halifax, NS B3H 4R2 Canada

**Keywords:** Systems analysis, Biological physics, Biomarkers

## Abstract

Using longitudinal study data, we dynamically model how aging affects homeostasis in both mice and humans. We operationalize homeostasis as a multivariate mean-reverting stochastic process. We hypothesize that biomarkers have stable equilibrium values, but that deviations from equilibrium of each biomarker affects other biomarkers through an interaction network—this precludes univariate analysis. We therefore looked for age-related changes to homeostasis using dynamic network stability analysis, which transforms observed biomarker data into independent “natural” variables and determines their associated recovery rates. Most natural variables remained near equilibrium and were essentially constant in time. A small number of natural variables were unable to equilibrate due to a gradual drift with age in their homeostatic equilibrium, i.e. allostasis. This drift caused them to accumulate over the lifespan course and makes them natural aging variables. Their rate of accumulation was correlated with risk of adverse outcomes: death or dementia onset. We call this tendency for aging organisms to drift towards an equilibrium position of ever-worsening health “mallostasis”. We demonstrate that the effects of mallostasis on observed biomarkers are spread out through the interaction network. This could provide a redundancy mechanism to preserve functioning until multi-system dysfunction emerges at advanced ages.

## Introduction

Homeostasis is the self-regulating process that maintains internal stability^1^. Yet as individuals age, it is characteristic for biomarkers to drift away from healthy levels; something about homeostasis is therefore “lost” during the aging process^2^. For example, loss of protein homeostasis is believed to cause the hallmark accumulation of unfolded, misfolded and aggregate proteins with age^3^. Accumulation is observed at multiple biological scales, including oxidative damage^4^, epigenetic age^5^, senescent cells^6^, and regulatory T-cells^7^ at the cellular scale, and extending up to the whole organism scale where clinical deficits^8^, including chronic diseases^9^, accumulate with age. Sehl and Yates performed univariate analysis of 445 health biomarkers and found that almost all of them accumulate negatively with age—typically showing linear decline^10^. Such accumulation of biomarker values in a particular direction appears to be a generic feature of aging. When biomarkers reach abnormal values, they are associated with dysfunction and poor health, independently of age^11,12^. A general mechanism of how accumulation and poor health emerge from homeostasis has, nevertheless, been missing.

Prior work suggests that accumulation may be a consequence of a drifting equilibrium position. Allostasis, literally “homeostasis through change”^13^, describes a version of homeostasis in which the equilibrium position is mutable, adapting as necessary to environmental demands^14^. Over time, “wear-and-tear” of this adaptive stress-response causes a subclinical accumulation of dysfunction known as “allostatic load”^14^. We hypothesize that these allostatic changes may be asymmetric, causing a coherent, population-level drift in equilibrium biomarker values with age, and ultimately leading to accumulating biomarker values in particular directions.

Directly estimating an individual’s allostatic load remains an open challenge^14^, owing to the confounding effects of the underlying interaction networks^12^. Instead, most algorithms infer allostatic load by outlier detection^12,14^ or other symmetric indicators, agnostic to any preferred biomarker accumulation direction^15,16^. These approaches have not been reconciled with generic, age-associated biomarker accumulation, which proceeds in preferred directions^10^. It therefore remains unclear how allostatic load leads to worsening health. Other theories posit that outlying biomarker values indicate damage, which promotes further damage e.g. as quantified by the number of health deficits (“frailty index”, FI)^11,17,18^. Needed is direct evidence of allostasis and how it is associated with worsening health.

Instability is another mechanism for accumulation. While linear accumulation is the norm^10^, some biomarkers accumulate exponentially with age e.g. senescent cells^6^ and the FI^8^. Exponential growth indicates an instability^19^. Nevertheless, a weak instability can appear linear until advanced ages. As a result, it remains unclear whether age-related accumulation proceeds due to a shifting equilibrium, a weak instability, or some hybrid of the two.

Operationalizing and quantifying homeostatic changes is challenging^14^ because homeostasis is a property of the whole system, not individual constituent parts^1,12^. In the language of complexity science^20^, homeostasis is an emergent property of a network of interacting variables. Each variable measures a part of the system, but changes to one part can be balanced by other parts. For example, heart rate declines with age but can be compensated for by increased stroke volume^10^ in order to maintain arterial blood pressure^1^. The essential aspects of homeostasis are: (i) a multivariate interacting dynamical system, (ii) an equilibrium state, which may vary with age (allostasis), (iii) the system spontaneously returns to the equilibrium state (dynamical stability), and (iv) stresses (and interventions) provide random shocks to the system. Altogether, homeostasis can be operationalized as a multivariate, mean-reverting stochastic process^16^.

Dynamical stability analysis uses eigen-decomposition to probe the stability of arbitrary systems^21,22^. The system is first linearized around an equilibrium position^21^. Orthogonal eigenvectors are then identified that decouple the interactions between variables. Eigenvectors are composite health measures that serve as *natural variables* since they do not interact or compensate for each other, and so can be analyzed individually. Each such natural variable has an associated eigenvalue that determines its stability via a characteristic recovery rate or timescale ($-\text {eigenvalue}=\text {rate}= \text {timescale}^{-1}$). A system is stable if and only if all recovery rates are positive^21^. Conversely, dynamical instability arises only if at least one recovery rate is negative.

We confront homeostasis with minimal assumptions. We seek generic changes to biomarker equilibrium and stability within aging organisms. We investigate multiple longitudinal datasets with multiple organisms (mice and humans) and multiple outcomes (dementia and death). In contrast to earlier work by Sehl and Yates^10^, our model is multivariate and generic so that we can model homeostasis without constraining its dynamical behaviour. We find that allostatic drift is consistent with the observed data. Importantly, we find that a small set of natural variables drive mortality and can be used to characterize an individual’s health state. We do not observe any dynamical instabilities.

## Model

To analyse stability for deterministic^21^ or stochastic^22^ dynamics, we use a linear approximation near a stable point,

1 $$\begin{aligned} \vec {y}_{i n+1}&= \vec {y}_{i n} + \varvec{W}\Delta t_{i n+1}(\vec {y}_{in} - \vec {\mu }_{in}) +\vec {\varepsilon }_{i n+1}, \nonumber \\ \vec {\varepsilon }_{i n+1}&\sim \mathcal {N}(0,\varvec{\Sigma }|\Delta t|_{i n+1} ) \nonumber \\ \vec {\mu }_{in}&\equiv \vec {\mu }_{0}+\varvec{\Lambda }\vec {x}_{in}+ \vec {\mu }_{age} t_{in} \end{aligned}$$

where *i* indexes the individual, *n* indexes the timepoint, $t_n$ is the age, $\vec {y}$ is a vector of observed biomarkers, and $\vec {x}$ is a vector of covariates that includes sex. $\varvec{W}$, $\varvec{\Sigma }$, and $\varvec{\Lambda }$ are constant matrices, independent of *i* and *n*. If we take the average over individuals (indicated by angled brackets) then we can obtain rates of average change

2 $$\begin{aligned} \frac{\langle \Delta y_{ij n+1} \rangle }{\langle \Delta t_{in+1} \rangle }&= W_{jj}\langle y_{ijn} - \mu _{ijn} \rangle + \sum _{k\ne j} W_{jk}\langle y_{ikn} - \mu _{ikn} \rangle . \end{aligned}$$

We see that changes to the mean of a particular biomarker, $y_j$, are due either to recovery of $y_j$ towards the equilibrium position, $\mu _j$, or because of interactions with a compensating variable, $y_{k\ne j}$ through off-diagonal elements of $\varvec{W}$. This provides both a mechanism for biological redundancy—if the organism can actively influence some of the $y_k$ then it can use them to steer others—and a mechanism for mutual dysfunction—since $\varvec{W}$ couples dysregulation of $y_{k\ne j}$ to that of $y_j$. In Supplemental Sect. S8 we show that Eq. (1) approximates general nonlinear dynamics^21^. We also specifically show that it approximates the stochastic process model^16^, a framework for aging biomarker dynamics. Note that the model permits unequally-spaced sampling of individuals through $\Delta t_{in+1}$, which is the time between measurements of individual *i* at time $t_n$ and $t_{n+1}$.

**Figure 1:**
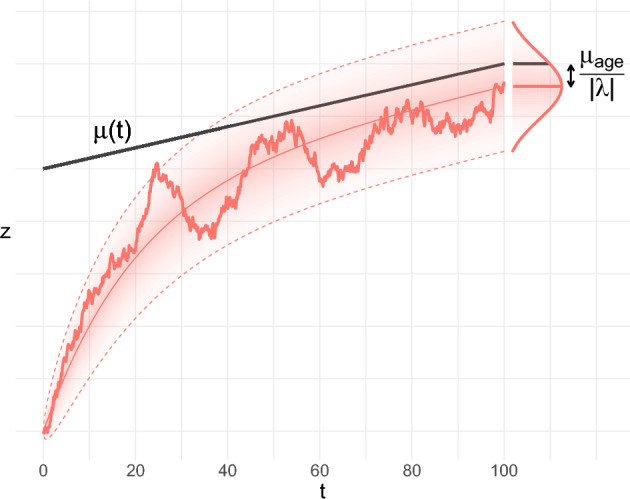
Simulation example of a stable system, with $\lambda < 0$. Initial conditions can differ from $\mu (t)$. A stable system is attracted to $\mu (t)$ (black line), but will be offset by $-\mu _{age}/|\lambda |$ in the steady-state. ODE solutions are superimposed for mean and variance (dotted lines are 95% interval). Fill density is proportional to probability density. Observing an ensemble at any time will yield Gaussian statistics.

The stability of the model depends on the eigenvalues of $\varvec{W}$. We can decouple variable means with the eigenvector transformation matrix $\varvec{P}$. We obtain

3 $$\begin{aligned} z_{ijn+1}&= z_{ijn} + \lambda _j\Delta t_{in+1}(z_{ijn} - \tilde{\mu }_{ijn}) +\tilde{\varepsilon }_{ij}, \end{aligned}$$

where $\vec {z}_n\equiv \varvec{P}^{-1}\vec {y}_n$, $\lambda _j \equiv P_{j\cdot }^{-1} \varvec{W} P_{\cdot j}$, $\tilde{\vec {\mu }}_{n} \equiv \varvec{P}^{-1}\mu _{n}$ and $\tilde{\vec {\varepsilon }} \equiv \varvec{P}^{-1}\vec {\varepsilon }$. We refer to $\vec {z}$ as *natural variables*. The natural variables build correlations only through the noise term, $\tilde{\varepsilon }$—in addition to any correlated initial conditions. The system is mutually-diagonal if $\tilde{\varepsilon }$ is uncorrelated. While our dynamics are discrete, it is also helpful to consider continuous dynamics corresponding to the limit $\Delta t \rightarrow 0$; see Fig. 1 and Box 1 (also Supplemental Sect. S8 for more details).

The parameters $\varvec{W}$, $\vec {\mu }_0$, $\vec {\mu }_{age}$ and $\varvec{\Lambda }$ are estimated from the data ($\varvec{\Sigma }$ can also be). The stochastic term, $\vec {\varepsilon }$, is assumed to be normally distributed and independent across timepoints. See Supplemental Sect. S6 for details. (For the remainder of the paper, we simplify notation by dropping the tilde and suppressing the individual *i* and timepoint *n* indices.) Optimal parameter values are selected by maximizing the likelihood. For uncorrelated noise this reduces to weighted linear regression.

If a mutually-diagonal system reaches steady-state—having run long enough to forget initial conditions—then the natural variables, $\vec {z}$, are the principal components, ranked by stability (Supplemental Eq. (S49)). We used principal component analysis (PCA) as a preprocessing step. If $\varvec{P}$ is orthogonal (which it is from PCA) then Parseval’s theorem states that $\langle \sum _j y_{jn}^2 \rangle = \langle \sum _j z_{jn}^2 \rangle = \sum _j ( \text {Var}(z_j) + \langle z_j \rangle ^2)$: this means that a single $z_{k}$ with large mean and variance can dominate that of the $\vec {y}$.

Box 1: Ordinary differential equation (ODE) behaviour
Consider a 1-dimensional space, *z*. If we take the limit $\Delta t\rightarrow 0$ then Eq. (3) is a modified Ornstein-Uhlenbeck process. The mean and variance are solutions to ordinary differential equations. The mean is described by

4 $$\begin{aligned} \frac{d}{dt} \langle z \rangle = \lambda (\langle z \rangle - \mu (t)) = \lambda \langle z \rangle - \lambda \mu _0 - \lambda \mu _{age} t \end{aligned}$$

where $\mu _{age} t$ is the time-dependent part of $\mu _n$ and $\mu _0$ is the remaining part. The general solution of Eq. (4) is

5 $$\begin{aligned} \langle z \rangle (t)&= \big (\langle z_0 \rangle - \frac{\mu _{age}}{\lambda } - \mu _0\big )e^{\lambda t} + \frac{\mu _{age}}{\lambda } + \mu _0 + \mu _{age} t \end{aligned}$$

where $\langle z_0 \rangle$ is the initially observed mean at $t=0$. The exponential factors dampen or aggregate the mean depending on the sign of $\lambda$. If $\lambda < 0$ the system is stable and once $|\lambda | t \gg 1$ a dynamical steady-state (ss) is reached,

6 $$\begin{aligned} \langle z \rangle _{ss}(t)&= \frac{\mu _{age}}{\lambda } + \mu _0 + \mu _{age} t = \mu (t) - \frac{\mu _{age}}{|\lambda |}. \end{aligned}$$

The steady-state is equivalent to the system forgetting its initial conditions. This steady-state behaviour can explain the drift observed by Sehl and Yates^10^ (Supplemental Sect. S8.6). In the steady-state, the mean drifts at a constant rate,

7 $$\begin{aligned} \frac{d}{dt}\langle z \rangle _{ss}(t)&= \mu _{age}, \end{aligned}$$

but there is a constant lag of $\langle z - \mu \rangle _{ss} = \mu _{age}/\lambda$; Fig. 1 illustrates. Only when $\mu _{age}=0$ (no drift) is $\mu (t)$ the steady-state position. Outside of steady-state, the mean is displaced by

8 $$\begin{aligned} \langle z - \mu \rangle (t)&= \langle z - \mu \rangle (t_0) e^{\lambda (t-t_0)} + \frac{\mu _{age}}{\lambda }(1-e^{\lambda (t-t_0)}) = \text {Memory} + \text {Drift} \end{aligned}$$

for reference time $t_0$. The first term encodes the system’s initial conditions, whereas the last term encodes long-time drifting behaviour. Systems near steady-state exhibit $\text {Memory} \ll \text {Drift}$.

If $\lambda =0$ the system is marginally stable and preserves its initial conditions. If $\lambda >0$ the system is unstable and the initial conditions grow exponentially over time. In either case the steady-state is never reached.

In contrast to the mean, for $\lambda < 0$ the variance eventually equilibrates, reaching a constant value. The variance is described by

9 $$\begin{aligned} \frac{d}{dt} \text {Var}(z) = 2\lambda \text {Var}(z) + \sigma ^2 \end{aligned}$$

where $\sigma ^2$ is the noise strength. The general solution is given by

10 $$\begin{aligned} \text {Var}(z)(t) = \text {Var}(z_0)e^{2\lambda t} - \frac{\sigma ^2}{2\lambda }( 1 - e^{2\lambda t} ) \xrightarrow [\lambda < 0,~t \rightarrow \infty ]{\text {steady state}} \frac{\sigma ^2}{2|\lambda |}. \end{aligned}$$

Approaching instability, with $\lambda \rightarrow 0$, the system accumulates noise.

## Results

We analysed four datasets originating from three studies: two human and two mouse. Our analysis focused on the key properties of homeostasis: stability and equilibrium position. We used model selection to compare our model to the null hypothesis and to pick an optimal model form (Supplemental Sect. S5).

We observed that both an interaction network, $\varvec{W}$, and an equilibrium term, $\vec {\mu }$, were needed to optimally predict future biomarker values. We saw no evidence of nonlinear terms in the dynamics. We found that fitting Eq. (3) using principal components (PCs) yielded equivalent performance to the model with full flexibility, Eq. (1), but was already in diagonal form. Hence for each dataset we analysed a set of decoupled, one-dimensional equations in $z_j$ (with *j* sorted by stability, so that $j=1$ is the least stable in each study).

For covariates, we generally found non-significant improvements in prediction—though we kept them to improve interpretability (to reduce confounding). The exception was the age covariate, $\mu _{age}$, which significantly improved the fit of the SLAM Het3 mice (SLAM C57/BL6 were almost significant). The presence of $\mu _{age}$ indicates allostasis in the form of a time-dependent homeostasis.

**Figure 2:**
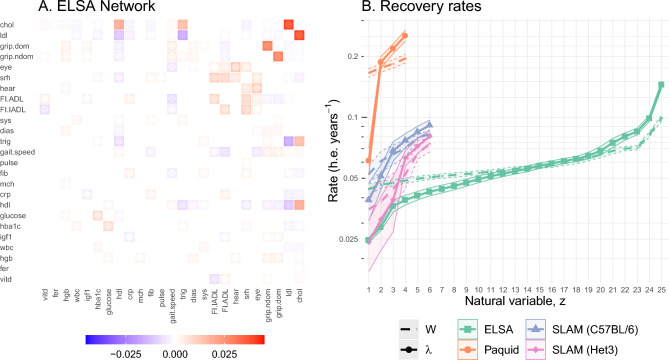
(**A**) ELSA interaction network. Tile colour indicates interaction strength (saturation) and direction (colour) of the interaction from the y-axis variable to the x-axis variable. Inner dot colour indicates the limit of the 95% confidence interval (CI) closest to zero (more visible point indicates lower significance). Non-significant interactions have been whited-out. Diagonal has been suppressed for visualization (see dotted lines in **B**). The matrix is real and symmetric because the data were diagonalized by an orthogonal matrix (PCA). Variables are sorted by diagonal strength in both A. and B. (increasing rate). (**B**) Recovery rates in human-equivalent (h.e.) years i.e. negative eigenvalues ($-\lambda$). The smallest recovery rates determine system stability^21^. A recovery rate of 0.025 implies $1-e^{-1}=63\%$ recovery after $-\lambda ^{-1}=40$ years (95% recovery after 120 years). The survival data all have similar minimum rates near 0.025, whereas the dementia data was faster (Paquid). The dotted lines are network diagonals ($-W_{jj}$); the solid lines are rates ($-\lambda _j$).

The interaction networks between variables can be represented by the respective weight matrices, e.g. Fig. 2A. For ELSA we see expected relationships, e.g. total/HDL/LDL cholesterol, and non-dominant/dominant grip strength. ELSA also shows a block of like-variables including the FI-ADL, FI-IADL, self-reported health (srh), and gait speed, which could relate to frailty^23^. See Supplemental Fig. S9 for networks from the other datasets.

The interactions between observed biomarkers prevents us from assessing stability directly. However, we can eigen-decompose the networks to yield an equivalent non-interacting network of natural variables. Each natural variable has a characteristic recovery rate (Fig. 2B). All natural variables were stable, with $\lambda <0$. Faster recovery rates indicate higher stability (resilience)^22^. It takes 3 timescales for the system mean to recover 95% of the way to equilibrium. For each mortality dataset the recovery timescale of its slowest natural variable was comparable to the organism’s lifespan, $\approx 40~$ human-equivalent years; only the mental acuity dataset (Paquid) was faster ($\approx 20$ years). In all datasets the rates for the natural variables extended to higher and lower values than the diagonal elements of the observed biomarkers (compare solid to dotted lines)—indicating that network interactions play an important role in recovery dynamics.

**Figure 3:**
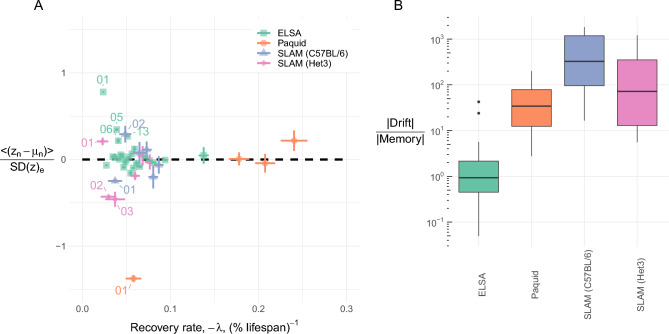
(**A**) Position relative to equilibrium vs recover rate. Most natural variables were homeostatic (near equilibrium at 0). Some (labeled) variables were observed to be far from equilibrium; variables are labelled by rank e.g. $01 \equiv z_{01}$ has the fastest recovery (furthest left). (**B**) Characterization of natural variable deviations from equilibrium using Eq. (8). Observe that ELSA is the only dataset where memory may dominate the system behaviour (ratio $\lesssim 1 = 10^0$), indicating that the followup period may have been too short to reach a steady-state. In both figures only mouse (SLAM) data points over age 80 weeks were used since biomarkers had a u-shaped curve over the lifespan^24^.

We summarize homeostasis in Fig. 3A, using the population means. If variables are in homeostasis then the mean should be close to $\mu _n$, where the scale is determined by the native dispersion. Each dataset had most natural variables near μ_n_ with a few outliers, such as $z_1$ for all datasets. (In contrast, the majority of observed biomarkers had large deviations from equilibrium—see Supplemental Fig. S10). We characterized the natural variable dynamics using Eq. (8) in Fig. 3B. Excluding ELSA, most data points were in a steady-state, as indicated by their small memory term (relative to drift). The steady-state mean includes a drift caused by $\mu _{age}$. Across variables, the deviations from equilibrium observed in Fig. 3A, $\langle z_n - \mu _n \rangle$, were very strongly correlated with $\mu _{age}$, with correlation coefficients: − 0.988 ($p=2\cdot 10^{-4}$, SLAM BL/6), − 0.947 ($p=10^{-3}$, SLAM Het3), − 0.989 ($p=0.01$, Paquid), and − 0.302 ($p=0.14$, ELSA). This is consistent with Eq. (6), and supports our use of an allostatic model with equilibrium drifts given by $\mu _{age}$. The smaller correlations observed with the ELSA dataset are consistent with the strong memory effect seen in Fig. 3B—violating the steady-state assumption of Eq. (6). ELSA may have failed to reach steady-state due to the limited followup period, which was the shortest of all datasets by a factor of 2, or could indicate the confounding effects of medical interventions, which are not relevant for the other datasets.

**Figure 4:**
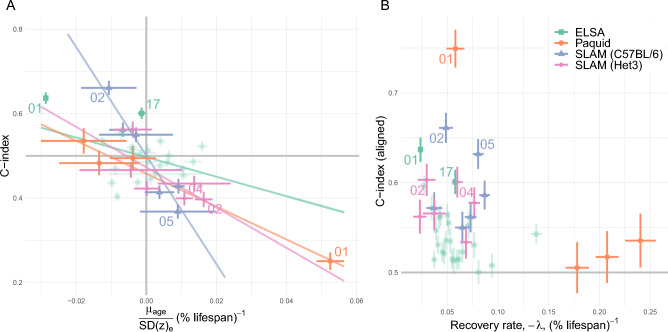
Survival effects. (**A**) Allostasis drifts towards the risk direction, i.e. “mallostasis”. The relationship appears to be linear (lines), with strong correlations: − 0.96 (SLAM BL/6), − 0.71 (SLAM Het3), − 0.99 (Paquid), and − 0.53 (ELSA). The equilibrium dispersion provides a native scale for each variable. High risk natural variables for each dataset have been labelled by eigenvalue rank (e.g. $z_1 \equiv 01$ has the smallest eigenvalue, $z_2 \equiv 02$ the second smallest, etc). (**B**) Recovery rate (-eigenvalue), $-\lambda$, has an ambiguous relationship with survival. Smaller eigenvalues appear to be important survival dimensions (e.g. 01 for ELSA and Paquid), but the overall correlation is weak ($\rho =-0.254$, $p=0.1$). The C-index measures the relative risk for pairs of individuals based on the value of $z_j$ (C-index of 0.5 indicates no risk; C-index larger than 0.5 means small values are bad).

Most natural variables have small drift and are effectively homeostatic—with only a few strongly drifting allostatic natural variables. The steady-state drift rate of natural variables, $\mu _{age}$, was correlated with the survival risk for each dimension (Fig. 4A). The correlations were typically strong: − 0.958 ($p=0.002$, SLAM BL/6), − 0.713 ($p=0.1$ SLAM Het3), − 0.987 ($p=0.01$, Paquid), and − 0.534 ($p=0.006$ ELSA); overall: − 0.742 ($p=3\cdot 10^{-8}$). The correlation was weakest for ELSA, which had not reached steady-state. The Cox proportional hazards coefficients, conditioned on age and sex, showed a similarly strong correlation with $\mu _{age}$, 0.70 ($p=10^{-7}$, all data) (Supplemental Fig. S14). Furthermore, we see that the drift direction, $\text {sign}(\mu _{age})$, is the same as the risk direction ($p=0.0003$, Fisher test). Hence, not only does homeostasis drift with age, the direction of the drift is *towards ill-health*. The primary risk directions were $z_1$ for ELSA and Paquid and $z_2$ for SLAM. Interestingly, $z_2$ of the Het3 mice is nearly identical to $z_1$ of the C57BL/6 mice in terms of covariates and survival effect—hence $z_1$ of the C57BL/6 is also likely a key risk direction (Supplemental Figs. S11 and S18). Regardless, $z_1$ or $z_2$ exhibited the strongest survival effect for each dataset (Fig. 4A). These variables also both had small eigenvalues ($z_1$ is rank 1 and $z_2$ is rank 2). However, this relationship between survival and eigenvalue magnitude does not appear to generalize, see Fig. 4B.

**Figure 5:**
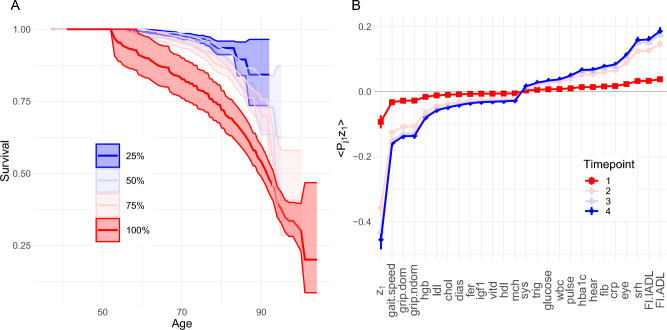
(**A**) Composite health measure of survival $b\equiv (\vec {\mu }_{age}^T\vec {z})$, stratified by quartile (ELSA). Separation is excellent, indicating a strong survival predictor. Fill is 95% confidence interval. See Supplemental Fig. S15 for the other datasets. (**B**) Natural variables can drive changes in observable biomarkers. The $z_1$ mean is accumulating in the negative direction. This accumulation is mapped into observable variables with $\langle P_{j1} z_1 \rangle$ for indicated timepoints each separated by approximately 4 years. The drift direction is overwhelmingly unhealthy: increased disability measures (srh, eye, hear, FI.ADL and FI.IADL—high is bad), decreased physical ability scores (gait and grip), increased inflammation (crp), increased glucose, etc. The effect of the drift is concentrated in $z_1$ but dilute across its covariates, which could make the effect of unhealthy $z_1$ subclinical in the observed biomarkers. All variables are on standardized scale. Similar effects were observed for the other datasets (Supplemental Fig. S13).

As an illustration of the utility of the correlation between survival and $\mu _{age}$, we consider a simple summary health measure. The Cox proportional hazards model assumes the hazard scales as $\exp {(\vec {\beta }^T\vec {z})}$, where the *j*th coefficient, $\beta _j$, is the log–hazard ratio per unit increase of $z_j$. As mentioned in the previous paragraph, $\vec {\beta } \sim \vec {\mu }_{age}$ were correlated; the relative hazard can therefore be approximated by $\exp {(\vec {\mu }_{age}^T\vec {z})}$. Indeed, we observed that $b\equiv \vec {\mu }_{age}^T\vec {z}$ is an excellent predictor of survival, see Fig. 5A and Supplemental Fig. S15.

The natural variables with large $|\mu _{age}|$ will eventually experience the largest drift, according to Eq. (7). $z_1$ in Fig. 5B is an example of such an accumulating variable. The other variables with large $|\mu _{age}|$ experienced a similar accumulation (Supplemental Fig. S13). For an orthogonal transformation such as $\varvec{P}^{-1}$, the sum of the variance and squared mean is conserved (Parseval’s theorem). Natural variables with large means and variances will therefore disproportionately affect the means and variances of observed biomarkers. The effect is demonstrated for ELSA in Fig. 5B. As the dominant natural variable drifts it influences observable biomarkers to drift as well.

Slower recovery rates (eigenvalues) take longer to forget perturbations, causing the associated natural variables to accumulate variance due to noise. Recall that the slowest recovery rates were on the order of a lifespan (Fig. 2B). The Pearson correlations between the estimated variance and rate (-eigenvalue) were strong: − 0.852 ($p=0.03$, SLAM BL/6), − 0.802 ($p=0.05$ SLAM Het3), − 0.998 ($p=0.002$, Paquid), and − 0.764 ($p=9\cdot 10^{-6}$ ELSA) (log–log scale; see Supplemental Fig. S16). Hence the variances we observe at old ages will be dominated by the variables with the smallest eigenvalues, $\lambda$ (e.g. $z_1$ and $z_2$). As we have seen before, these variables are often—but not always—strongly associated with adverse effects, depending on the drift rate $\mu _{age}$. This suggests that most of the age-related changes to health were concentrated in a few $z_k$ which drive both biomarker drift (mean) and dispersion (variance). Growing variance along these dimensions may capture individual accumulation of stochastic damage, such as genetic damage or disease.

## Discussion

We fit a homeostasis model of equilibrium and stability to four longitudinal aging datasets (two mouse and two human) using generic health biomarkers. Our model is lightweight, can be estimated using standard statistical algorithms, and is sufficient to capture essential information about the aging process. Health biomarkers have an equilibrium position, $\vec {\mu }$. Their corresponding stochastic term, which has covariance $\varvec{\Sigma }$, represents random stresses that drive individuals away from equilibrium; as well as residual effects such as individual variability and nonlinearities. An interaction network, $\varvec{W}$, pulls individuals towards equilibrium either through recovery (diagonal terms) or by one variable compensating for another (off-diagonal terms). By eigen-decomposing $\varvec{W}$ we transformed the dataset into non-interacting, natural variables—which are linear combinations of the input biomarkers. This increases interpretability and simplifies analysis. The stability of the system is described by the recovery rates of the natural variables—which are the corresponding eigenvalues with flipped signs, $-\lambda$.

We modelled homeostasis as stability around an equilibrium mean value. Stability can only be assessed using the natural variables because the original biomarkers have interactions between them. Homeostasis was violated by some natural variables (Fig. 3A). Although most natural variables had average values near the homeostatic equilibrium—indicative of homeostasis—several were far away. We determined that this latter group were out of homeostasis because they were chasing drifting equilibrium positions from behind. This equilibrium drift with age represents allostasis, i.e. a changeable equilibrium position.

Allostatic variables accumulated over the course of the study period as they chased after the drifting equilibrium, systematically increasing or decreasing. This was facilitated by an age-dependent equilibrium position, governed by $\mu _{age}$, and was typically accompanied by a small eigenvalue. The gap between the population and allostatic equilibrium position is governed by $\mu _{age}/\lambda$, so a small $\lambda$ enables a large gap such that the entire population drifts coherently towards the moving equilibrium—causing population-level accumulation. This makes the linear drift term, $\mu _{age}$, the primary culprit for causing biomarkers to drift with age. Presumably $\mu _{age}$ arises from either (a) the effects of unknown biomarkers/mechanisms not included in the model (i.e. $\mu _{age} t \approx \sum _{k\ne j} W_{jk}\langle y_{ikn} - \mu _{ikn} \rangle$ in Eq. (2) for a set of unknown $y_{ikn}$), or (b) asymmetric stressors, which cannot be captured by our symmetric stochastic term (e.g. there is no such thing as negative damage so health deficits skew positive^8^).

The transformation to natural variables effectively compressed the drifting (accumulating) mean of many variables into a small number of natural variables. The natural variables can be thought of as the underlying cause of the observed biomarker drift (Fig. 5B). In this manner, the widely observed age-related decline in biomarkers^10^ are governed by a few natural variables—which are not directly observed. The effect is spread out by the transformation, potentially hiding the observed biomarker decline below diagnostic thresholds. This may be a redundancy mechanism: the network permits the biological system to spread out the age-related decline to keep biomarkers in healthy ranges for longer. The trade-off may be that many biomarkers would reach unhealthy ranges concurrently, leading to multisystem dysfunction. For example, the effects of chronic kidney disease are mild and non-specific until the patient nears kidney failure—at which point multisystem failure is imminent, typically leading to death via cardiovascular disease^25^. This tradeoff assumes that diagnostic thresholds represent critical values beyond which deterioration of a biological system accelerates. Univariate dynamical modelling of senescent cell count in mice^6^ and *E. coli* membrane integrity^26^ supports the existence of such critical values, where repair mechanisms saturate and decline accelerates. From this perspective, an individual’s robustness would depend on their buffer space available to absorb new insults, which could be quantified by the natural variable scores together with the stressor effect strength which should be proportional to the noise $\sigma$.

Consistent with this perspective^6^, the allostatic drift rate, $\mu _{age}$, strongly correlated with the mortality/dementia risk associated with each natural variable (Fig. 4A and Supplemental Fig. S14). Since $\mu _{age}$ is the steady-state drift rate of the mean, the steady-state behaviour is continually worsening health due to the drifting mean. Prior work on operationalizing allostasis has neglected the existence of a preferred risk direction, instead using the absolute distance from allostasis as a mortality factor^12,15,16^, irrespective of whether biomarkers are high or low. In contrast, our results indicate that numerous natural variables do, in fact, have preferred risk directions. Aging researchers should be aware of this symmetry breaking. This means that the adaptive changes due to allostasis at best mitigate declining health and, at worst, lead to a further decline in health. We refer to this phenomenon as “mallostasis”: the tendency of an aging biological system towards an ever-worsening equilibrium position. We have used this phenomenon both to identify important survival variables and to generate a novel composite health measure.

Our key quantitative results coincide with three key qualitative predictions made by allostatic load theory: (i) a shifting equilibrium position for biomarkers indicative of adaptive changes (allostasis, Fig. 3A), (ii) the shift is associated with adverse outcomes (mallostasis, Fig. 4A), and (iii) the shift is subclinical due to compensating mechanisms between biomarkers (transformation, Fig. 5B)^14^. This is compelling evidence that allostasis is a generic aging phenomenon, rather than being specific to neuroendocrinology. Our proposed composite health measure is therefore a novel estimator of allostatic load. In contrast to conventional estimators^14^, we were able to estimate allostatic drift directly as $\mu _{age}$. Our results rely on using natural variables, which are canonical coordinates that greatly simplify analysis.

Allostatic load is believed to arise from the long-term costs of short-term protection against stressors^13^, making it an example of antagonistic pleiotropy^27^. Alternatively, long-term costs could reflect imperfect repair. Regardless, long-term costs that accumulate in a given direction would lead to the allostatic drift which we have observed and characterized. Furthermore, we observed slow dynamical rates for the dominant mortality-risk natural variables (Fig. 2B). Accordingly, the dynamical timescale of these effects are comparable to the organismal lifetime—consistent with long-term costs.

Interestingly, we did not observe any instabilities or nonlinearities. We had expected that “allostatic overload”—the final state of allostatic load theory^14^—would operationalize as an instability. However, instabilities, and their associated exponential growth, are rare among health biomarkers^10^; although they are observed in summary measures of health such as the FI (frailty index)^8^. Other unstable, FI-like variables can be extracted from generic biomarkers using nonlinear techniques such as a deep neural network^19^, diagnostic thresholds^11^, or quantile-based preprocessing^18,28^. Since we did not see evidence of instabilities or other nonlinearities in the natural variables, the nonlinear embedding or discretization should be considered as a possible cause for observed FI-like instabilities. It may be that biological systems naturally suppress nonlinear effects of aging—obscuring the effects—or, conversely, that aging is primarily a linear phenomenon that slowly pushes individuals towards nonlinear tolerance thresholds for dysfunction/damage, e.g. saturation of repair^6,26^ and/or emergence of chronic disease. A non-trivial issue is that exponential growth often appears linear, for example the FI in mice and younger humans ($\lesssim 85$ years old)^29^. Nonlinear effects in biomarker dynamics may require special populations, such as the ill or exceptionally old, to be observed.

The key model variables, $z_1$ and $z_2$, dominate the aging process. These natural variables with smaller $\lambda$ carried the majority of the variance and become the dominant principal components in the steady-state model (Supplemental Fig. S16 and Eq. (S49), respectively). Applying Parseval’s theorem, these variables will control the variance of directly observed biomarkers. Since they also dominate the means via allostatic drift, they will determine the aging phenotype that we observe. Both effects get stronger with age. This means that the empirically observed age-related changes in the mean and variance of biomarkers will be predominantly caused by only a few key natural variables. Hence the nearly-universal linear decline in health biomarkers observed by Sehl and Yates^10^ may simply be a few declining natural variables spread across the observed biomarkers (Supplemental Sect. S8.6). Furthermore, this implies that a single dimensional decline can drive many observed biomarkers, which is the foundational assumption of “biological age” estimators^30,31^. Our results provide much needed support for such low dimensional representations of aging—which should become increasingly accurate with advancing age since the means of the key natural variables grow fastest, and their variances grow largest.

The natural variables, *z*, should be good choices for targeting and monitoring interventions. They are prospective biomarkers with the convenient property that if you can intervene on one it will not affect the others. In contrast, we know from the network of interactions that intervening on any single biomarker is likely to affect many other biomarkers. In the steady-state, the mallostatic drift rate, controlled by $\mu _{age}$, is a proxy for the hazard and therefore identifies the most important targets of intervention. The coefficients of the transformation, $\varvec{P}$, provides both hints at what mechanisms each $z_j$ is capturing as well as a map for which biomarkers will be affected by interventions on $z_j$. For example, $z_1$ of ELSA shares many features with frailty: strong age dependence, large effect in gait, weakness (grip strength), disability and self-reported health, and large survival effect^23^. $z_1$ is thus a prospective biomarker of frailty and can be used both to monitor an individual’s frailty and to engineer interventions. The strong signals we see in Fig. 5B for gait, grip strength and activities of daily living are hints that loss of physical fitness is one mechanism by which frailty proceeds and therefore one mechanism by which we can intervene, consistent with a meta-analysis which has shown that physical activity can reduce frailty in humans^32^. Remarkably, we observed other prospective targets in addition to $z_1$ of ELSA. Given an organism and set of biomarkers, each $z_j$ with substantial drift should be considered a prospective intervention target, with the faster drifting being the most important.

We note a few limitations of our study. We assumed linear, time-invariant interactions through the network, $\varvec{W}$—following previous work that suggested that interactions are linear and time-invariant^33^ (as are the principal components^23^). The networks we extracted were symmetric and hence acausal due to our use of PCA as a preprocessing step, although we did estimate more general networks and found they performed no better. This could be a consequence of the data which were entirely observational, obfuscating causality. Understanding interventions using our results is similarly subject to the caveat that biomarkers behave the same whether they are observed or intervened upon^34^. This seems plausible since observational studies include everyday interventions such as disease, medicine, or lifestyle changes. Finally, our model is at the population-level and hence we cannot resolve homeostatic changes at the individual level.

We see exciting opportunities for future work. Our observation that principal components could be effectively used as independent variables suggests that more complex statistical models could also be applied. For example, individual-level model parameter estimates via mixed-effects modelling would help to determine whether individual health changes are gradual or sudden (or possibly critical^35^). Changes in our model parameters due to age, chronic or acute illness, or medical interventions is particularly interesting, but will require specialized datasets to assess. Fortunately, small datasets are tractable with our linearized model. The generic nature of the model and its ability to find accumulating natural variables could also be applied to other biological or temporal scales. Others have postulated that damage aggregates due to dysfunction in regulatory systems or other intermediate scales^3^, which could be tested. Composite health measures, including biological age^31^, are also interesting to explore using our approach. Applying our approach using multiple biological ages as biomarkers^5^ will naturally extract salient information regarding stability and mallostasis, as well as a smaller set of essential natural variables. New datasets will open up new opportunities for this analysis pipeline. It is interesting to consider leveraging the effect of the natural variables to intervene and observe in clever ways. For example, $z_1$ appears to be a biomarker of frailty, which affects both mental and physical health^32^, hence we could potentially intervene based on a physical mechanism but monitor using cognitive changes.

We have developed and applied a lightweight network model that includes the salient features of homeostasis: equilibrium values and recovery rates. Equilibrium values are allowed to drift, to accommodate allostatic changes. Across datasets and species we consistently observed that the linear decline of biomarkers with age was governed by a small set of accumulating natural aging variables. This accumulation can be described as mallostasis: homeostatic dysfunction and associated declining health. These variables appear to be important measures of age-related decline, including health and mortality. Their effects are spread out by a network of interactions, driving drift in the observed biomarkers, and potentially diluting and obfuscating the effects of age. We find that generic biomarkers spontaneously move towards an equilibrium position which is itself continuously drifting towards ill-health. Mallostasis is a generic feature of the aging process.

## Methods

### Materials

We used 4 longitudinal datasets originating from 3 studies (organism, primary outcome): Paquid (human, dementia)^36^, SLAM (mouse, death)^24,37^ and ELSA (human, death)^38^. We directly modelled biomarkers, $\vec {y}$, and included covariates, $\vec {x}$, in the homeostatic term, $\vec {\mu }$, using Eq. (1).

The Paquid dataset is a random subset of 500 humans (212 males and 288 females) from the Paquid prospective cohort study, enriched in dementia prevalence^36^. Age range: 66–95 years-old. Individuals were measured on average every 3.2 years for a maximum of 9 timepoints. We modelled four ordinal variables, including three measures of mental acuity: mini-mental state examination (MMSE), Benton visual retention test (BVRT) and Isaacs set test (IST), along with a self-reported depression score (CESD). We considered for covariates: sex, age and education level (completed primary vs not).

The Study of Longitudinal Aging in Mice (SLAM) includes two datasets, one for each mouse strain. Both include body composition measures and glucose serum at 12 week intervals starting at 7 weeks of age and continuing for the lifespan of each mouse^37^. Body composition and serum measurements were staggered and had to be imputed. Covariates included age and sex. We dropped 538/66,138 measurements that were recorded after death; ostensibly these were coding errors. After preprocessing, the first dataset included 608 C57BL/6 mice (303 male and 305 female) measured on average every 6.2 weeks for a maximum of 20 timepoints (every 4.9 human-equivalent years). C57BL/6 mice are genetically similar (inbred) and prone to lymphoma and metabolic dysfunction^39^. The second included 611 Het3 mice (304 male and 307 female) measured on average every 4.2 weeks for a maximum of 27 timepoints (every 3.6 human-equivalent years). Het3 mice are a genetically heterogeneous cross of four inbred mice (including C57BL/6)^39^. We converted to human-equivalent years using the ratio of median survival times of each strain to ELSA. Full details of the study are described elsewhere^24,37^.

The English Longitudinal Study of Ageing (ELSA) is a representative sample of English people mostly aged 50 and over^38^. We used physical functioning questionnaire data and blood tests for 9330 humans (4063 males and 5267 females), reported at 4 timepoints, each separated by approximately 4 years. Our choice of 25 variables includes frailty measures, cardiometabolic biomarkers, and immune biomarkers (Supplemental Table S1). We considered waves 2, 4, 6 and 8, since only these contained the full suite of biomarkers. Covariates included age and sex. We considered only individuals whom were present both in wave 2 and in subsequent waves, thus excluding new recruits. Despite the large number of individuals, ELSA appeared to have the worst quality data due to high individual heterogeneity and low number of timepoints.

### Data handling

All missing data were imputed. Dead individuals were also imputed, as it reduced bias due to mortality in simulated data (Supplemental Sect. S4.1). We compared several imputation strategies, including carry forward/back, multivariate imputation using chained equations (MICE)^40^, and using our model to impute the model mean. Ultimately, we used carry forward/back followed by the model mean, except for ELSA which used each individual’s mean biomarker value followed by the population multivariate normal mean then model mean. See Supplemental Sect. S4 for details.

Estimation of our model, Eq. (1), used Supplemental Algorithm S1, which iteratively ($\times 5$) applied the maximum likelihood estimator:

11 $$\begin{aligned} \varvec{\hat{\Lambda }}&=\langle |\Delta t_{in+1} |\vec {y}_{in}\vec {x}_{in}^T \rangle _{i,n} \big ( \langle |\Delta t_{in+1} |\vec {x}_{in}\vec {x}_{in}^T \rangle _{i,n} \big )^{-1} - \varvec{W}^{-1}\langle \text {sign}(\Delta t_{in+1})(\vec {y}_{in+1}-\vec {y}_{in})\vec {x}_{in}^T \rangle _{i,n} \big ( \langle |\Delta t_{in+1} |\vec {x}_{in}\vec {x}_{in}^T \rangle _{i,n} \big )^{-1} \end{aligned}$$

for $\varvec{\Lambda }$ (which includes $\mu _0$ through $x_0 = 1$), and

12 $$\begin{aligned} \varvec{\hat{W}}&= \langle \text {sign}(\Delta t_{in+1})(\vec {y}_{in+1}-\vec {y}_{in})(\vec {y}_{in}-\vec {\mu }_{in})^T \rangle _{i,n} \big ( \langle |\Delta t_{in+1}|(\vec {y}_{in}-\vec {\mu }_{in})(\vec {y}_{in}-\vec {\mu }_{in})^T \rangle _{i,n} \big )^{-1} \end{aligned}$$

for $\varvec{W}$, where the expectation values are to be taken over times, *n*, and individuals, *i*. For the diagonal models we instead used weighted linear regression. Missing values were imputed with the model prediction after each iteration (except ELSA). Estimators are described and validated in Supplemental Sects. S6 and S7, respectively. We used a time-dependent Cox model to assess survival. We assumed stepwise constant covariates via start-stop formatting^41^. All correlations are Pearson. All errorbars are standard errors unless stated otherwise.

### Model assessment

We simultaneously estimated both parameter uncertainty and model performance using the standard deviation from $100 \times$ repeat bootstrap resampling. We compared model performance using the root-mean squared error (RMSE) and mean absolute error (MAE). Both were estimated using out-of-sample bootstrap^42^. In validation tests we found that a simple 632 estimator i.e., $\text {RMSE}_{632} \equiv 0.632 \cdot \text {RMSE}_{\text {test}} + 0.368 \cdot \text {RMSE}_{\text {train}}$, provided a good estimate for the true values of both performance metrics (Supplemental Fig. S7). 0.632 is the expected fraction of unique individuals in each bootstrap^42^.

### Supplementary Information

Supplementary Information.

## Data Availability

All data used are publicly available. The SLAM datasets are available from a previous publication^24^. Paquid is available from a software package^36^. ELSA^38^ is available from the UK Data Service https://ukdataservice.ac.uk/. Software for fitting and simulating our model is available at https://github.com/GlenPr/stochastic_finite_model.
