# Supplementary material for: Network dynamical stability analysis reveals key “mallostatic” natural variables that erode homeostasis and drive age-related decline of health
Source: Sci Rep. 2023 Dec 13;13:22140. doi: 10.1038/s41598-023-49129-7 (PMC10719307; doi:10.1038/s41598-023-49129-7)
Supplement: Supplementary file 1 — Supplementary Information. [file 41598_2023_49129_MOESM1_ESM.pdf]

Network dynamical stability analysis reveals key "mallostatic" natural variables that erode homeostasis and drive age-related decline of health Supplemental Information

Glen Pridham[1,†] **and Andrew Rutenberg**[1,‡]

[1]Department of Physics and Atmospheric Science, Dalhousie University, Halifax, Nova Scotia, Canada, B3H 4R2

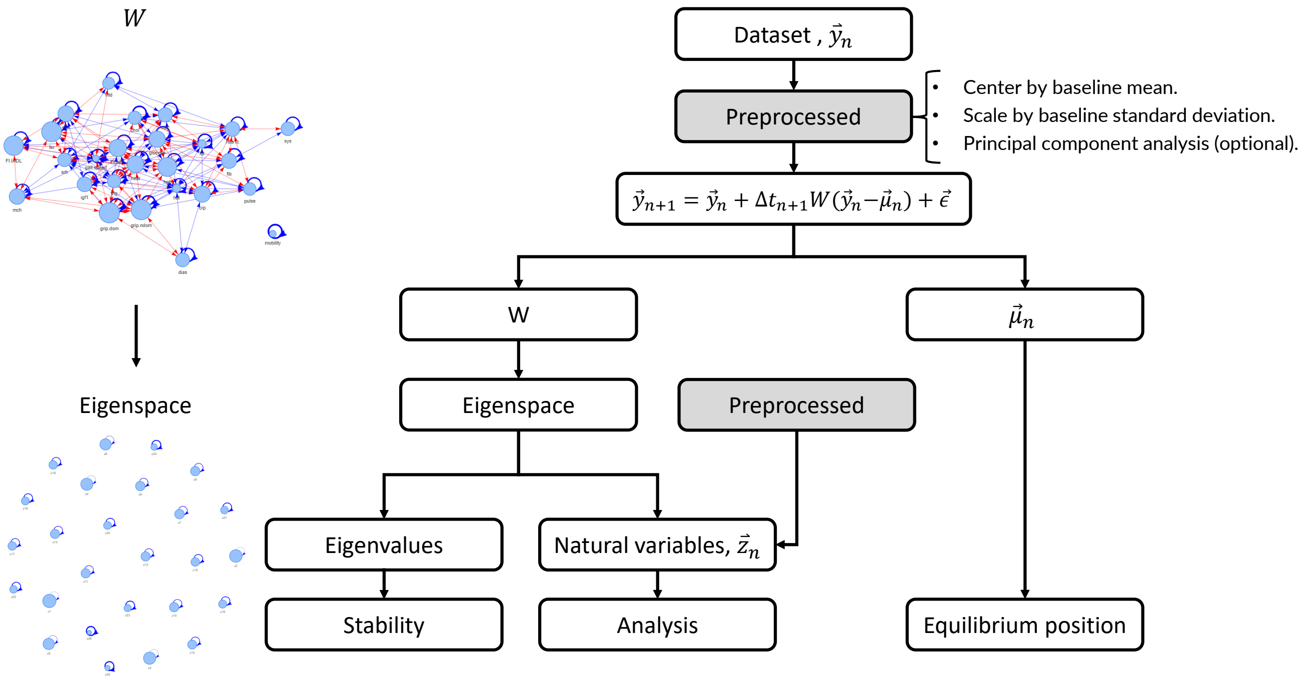

Figure S1. Study pipeline. We analysed four datasets using our proposed model. We model the dynamics of biomarkers, $\vec{y}_n$, over time using equation (S4). Our model extracts an interaction network, $\boldsymbol{W}$, and equilibrium positions $\vec{\mu}_n$, where the latter are allowed to depend on covariates (e.g. age and sex). The estimated network, $\boldsymbol{W}$, captures arbitrary linear interactions between biomarkers which can be removed by working with the natural variables, $\vec{z}_n$. Natural variables are defined by a linear mapping into the eigenspace of $\boldsymbol{W}$. The natural variables allowed us to analyse stability. We were also able to infer changes to the mean and variance of the observed variables based on changes in the natural variables.

S1 Introduction

We modelled generic health biomarker data as a mean-reverting stochastic process. Our study pipeline is summarized in Figure S1. Our model describes generic dynamics near an equilibrium solution (Section S8.4). In this supplemental we provide additional information to support and validate both our methods and our conclusions. We provide a complete description of the data in Section S2 and how we preprocessed it in Section S3. We performed a number of consistency checks on missing data which were imputed according to Section S4. We consider variations of our model in Section S5 which demonstrates that our final model best describes the data. We provide the mathematics necessary to estimate model parameters, along with an iterative estimation algorithm in Section S6. We then validate our algorithm using synthetic data in Section S7. Additional

mathematics useful for understanding our model and its connection to the literature are described in Section S8. Finally, we include additional results in Section S9 which support our conclusions.

Table S1. Dataset Summary — Biomarkers

Dataset	Species	Primary Outcome	Variable	Description
SLAM (C57BL/6)	Mouse, C57BL/6	Death	bw	Body weight
SLAM (C57BL/6)	Mouse, C57BL/6	Death	fat	Total fat mass
SLAM (C57BL/6)	Mouse, C57BL/6	Death	lean	Total lean mass
SLAM (C57BL/6)	Mouse, C57BL/6	Death	fluid	Total fluid mass
SLAM (C57BL/6)	Mouse, C57BL/6	Death	glucose	Blood glucose (fasting)
SLAM (C57BL/6)	Mouse, C57BL/6	Death	lactate	
SLAM (Het3)	Mouse, Het 3	Death	bw	Body weight
SLAM (Het3)	Mouse, Het 3	Death	fat	Total fat mass
SLAM (Het3)	Mouse, Het 3	Death	lean	Total lean mass
SLAM (Het3)	Mouse, Het 3	Death	fluid	Total fluid mass
SLAM (Het3)	Mouse, Het 3	Death	glucose	Blood glucose (fasting)
SLAM (Het3)	Mouse, Het 3	Death	lactate	
Paquid	Human	Dementia	MMSE	Mini-mental state exam†
Paquid	Human	Dementia	BVRT	Benton visual retention test
Paquid	Human	Dementia	IST	Isaacs set test
Paquid	Human	Dementia	CESD	Center for Epidemiological Studies depression scale‡
ELSA	Human	Death	vitd	Vitamin d
ELSA	Human	Death	srh	Self-reported health (higher: worse)
ELSA	Human	Death	eye	Self-reported (corrected) eyesight (higher: worse)
ELSA	Human	Death	hear	Self-reported (corrected) hearing (higher: worse)
ELSA	Human	Death	FI.ADL	Activities of Daily Living (ADL[1]) FI*
ELSA	Human	Death	FI.IADL	Instrumental ADL FI*
ELSA	Human	Death	gait.speed	Time to walk 8 feet (2.44 m)
ELSA	Human	Death	grip.ndom	Grip strength, non-dominant hand
ELSA	Human	Death	grip.dom	Grip strength, dominant hand
ELSA	Human	Death	crp	C-reactive protein⋆
ELSA	Human	Death	hba1c	Glycohaemoglobin
ELSA	Human	Death	glucose	Glucose
ELSA	Human	Death	hgb	Haemoglobin
ELSA	Human	Death	mch	Mean corpuscular haemoglobin
ELSA	Human	Death	fer	Ferritin⋆
ELSA	Human	Death	chol	Cholesterol
ELSA	Human	Death	ldl	Low density lipoprotein
ELSA	Human	Death	hdl	High density lipoprotein
ELSA	Human	Death	trig	Triglycerides⋆
ELSA	Human	Death	sys	Systolic blood pressure
ELSA	Human	Death	dias	Diastolic blood pressure
ELSA	Human	Death	pulse	Pulse
ELSA	Human	Death	fib	Fibrogen
ELSA	Human	Death	igf1	Insulin-like growth factor-1
ELSA	Human	Death	wbc	White blood cell count⋆

* FI: frailty index; defined as average number of health deficits[2].
† Transformed as $-\sqrt{\max(\text{MMSE}) - \text{MMSE}}$.
‡ Square root transformed for normality.
⋆ Log transformed for normality.

S2 Materials

We analysed 4 datasets derived from 3 longitudinal studies. The datasets and predictors ("biomarkers") used are summarized in Table S1. All predictor variables were continuous or, in the case of Paquid, ordinal with many scales (> 15).

We included covariates to reduce confounding effects and to look for allostasis, which depends on age. We included age (continuous) and binary variables. The covariates used are summarized in Table S2.

Table S2. Covariate Summary

Dataset	Covariate	Description
SLAM (C57BL/6)	Age	Chronological age in weeks
SLAM (C57BL/6)	Sex	0: male, 1: female
SLAM (Het3)	Age	Chronological age in weeks
SLAM (Het3)	Sex	0: male, 1: female
Paquid	Age	Chronological age in years
Paquid	Sex	0: male, 1: female
Paquid	Education	0: did not complete primary school, 1: did
ELSA	Age	Chronological age in years
ELSA	Sex	0: male, 1: female

S3 Preprocessing

The data we analyzed were longitudinal with regular sampling rates. For this reason, data were conveniently stored as 3-dimensional arrays (individuals, biomarkers, time points), meaning that each individual had the same number of variables and measurements (although many of them missing). This means that some timepoints for some individuals had to be 'invented' (instantiated as NA) based on the sampling rate of the study in question.

The Study of Longitudinal Aging in Mice (SLAM) datasets were both processed using the same criteria. The initial data were downloaded and processed using the analysis script of another publication[3]. We then applied additional preprocessing as follows. Biomarkers were visually investigated for normality and deemed adequate. The sex-specific mean and standard deviation of the first measurement of each biomarker was used to center and scale all timepoints. Mice with less than 2 timepoints were excluded from analysis (about 1% of mice). Any observations made past the reported death age of each mouse were excluded from analysis (about 1% of observations). We excluded the first two timepoints from analysis because after encoding we found that approximately half of individuals had not yet had a body composition measurement (imputed values looked unrealistic). Missing timepoints — which occurred due to staggered data collection — were instantiated using a piecewise linear model between known observations. Data from SLAM and the other datasets were stored in 3-dimensional arrays, with missing values imputed according to Section S4. The final arrays were size: (608, 6, 22) for SLAM C57/BL6, and (611, 6, 29) for SLAM Het3 (individuals, biomarkers, time points).

The Paquid dataset we used is available as part of a software package[4]. Biomarkers were visually investigated for normality. To improve normality we transformed CESD by the square-root and MMSE by $-\sqrt{30-\text{MMSE}}$ where 30 is the maximum allowed score for the MMSE. All biomarkers were centered and scaled by their respective mean and standard deviation from the first timepoint. Missing timepoints were instantiated using a piecewise linear model between known observations. The final array was size (500, 4, 9); (individuals, biomarkers, time points).

The English Longitudinal Study of Ageing (ELSA) dataset is available from the UK data service (https://ukdataservice.ac.uk/). We analysed all of the waves which included lab work: 2, 4, 6 and 8 (i.e. the "nurse" waves). We included only individuals present in wave 2, thus excluding later recruits. Biomarkers were visually investigated for normality. We found that the log transformation improved normality for C-reactive protein, ferritin, triglycerides, and white blood-cell count. All biomarkers were centered and scaled by their respective mean and standard deviation from the first timepoint. Skipped timepoints were instantiated using linear interpolation of the available timepoints. Censored (or died) timepoints were instantiated using the mean followup time (which was uniform due to the study design). The final array was size (9330, 23, 4); (individuals, biomarkers, time points).

S4 Missing data

We were presented with two forms of missing data for an individual at a particular timepoint. The entire timepoint could be missing or some subset of values could be missing. In either case the missingness could be informative; for example an individual may have temporarily left the study due to poor health and their biomarkers could have had abnormal values

reflecting their poor health. In this way the population may appear abnormally healthy as it ages. Under such circumstances, failure to impute can lead to biased study conclusions[5], such as parameter estimates (Section S4.1).

We considered three imputation approaches and selected the approach which gave the most reasonable values. First ("simplest"), we imputed a single value using either the individual's mean biomarker value, carry forward the previous value (and then carry back any skipped values), the conditional population mean (assuming multivariate Gaussian statistics), or the individual mean followed by the conditional mean for individuals whom did not have that variable reported. Second ("model mean"), we considered an iterative approach after applying one of the first methods wherein values were imputed according to the model mean (i.e. model prediction), equation (S1) and equation (S3). Third ("MICE"), we considered multivariate imputation using chained equations (MICE)[6]. MICE is a multiple imputation technique that uses a Gibbs' sampler along with a predictive model. We considered MICE using both classification and regression trees (CART) and 2-level modelling (normal for continuous variables and logistic for binary).

When imputing the model mean, at each iteration we estimated the model parameters then imputed the conditional mean for each missing value (Algorithm S1). Let $\vec{y}$ denote the biomarker, $\vec{u}$ denote all unobserved y and $\vec{o}$ denote all observed y. The statistics are Gaussian (equation (S4)), so we can use the factorization theorem[7] to compute the expectation value.

If $\vec{y}_{n+1}$ is known but a set of $\vec{y}_n$ are unknown then

$$\begin{aligned}
E(\vec{u}_n|\vec{o}_n,\vec{y}_{n+1}) &= \langle\vec{y}_{un}\rangle + \boldsymbol{\Sigma}_{uo}\boldsymbol{\Sigma}_{oo}^{-1}(\vec{o}_n - \langle\vec{y}_{on}\rangle) \quad \text{where,}\\
\langle\vec{y}_{un}\rangle &= (\boldsymbol{I}+\Delta t_{n+1}\boldsymbol{W})^{-1}_{u\cdot}\left(\vec{y}_{n+1}+\boldsymbol{W}\Delta t_{n+1}\vec{\mu}_n\right),\\
\langle\vec{y}_{on}\rangle &= (\boldsymbol{I}+\Delta t_{n+1}\boldsymbol{W})^{-1}_{o\cdot}\left(\vec{y}_{n+1}+\boldsymbol{W}\Delta t_{n+1}\vec{\mu}_n\right),\\
\Sigma_{uo} &= ((\boldsymbol{I}+\Delta t_{n+1}\boldsymbol{W})^T\boldsymbol{Q}(\boldsymbol{I}+\Delta t_{n+1}\boldsymbol{W}))^{-1}_{uo},\\
\Sigma_{oo}^{-1} &= \left(((\boldsymbol{I}+\Delta t_{n+1}\boldsymbol{W})^T\boldsymbol{Q}(\boldsymbol{I}+\Delta t_{n+1}\boldsymbol{W}))^{-1}_{oo}\right)^{-1},
\end{aligned} \tag{S1}$$

where $E(x|y)$ denotes expectation value of x conditional on y, $\boldsymbol{Q}$ is the precision matrix defined below, and $\boldsymbol{I}$ is the identity matrix. Note that

$$\boldsymbol{\Sigma} \equiv \boldsymbol{Q}^{-1} = \begin{bmatrix}\boldsymbol{\Sigma}_{oo} & \boldsymbol{\Sigma}_{ou}\\ \boldsymbol{\Sigma}_{uo} & \boldsymbol{\Sigma}_{uu}\end{bmatrix} \tag{S2}$$

is the block-decomposition of the noise covariance rearranged for observed (o) and unobserved (u) variables. If instead $\vec{y}_n$ is known but a set of $\vec{y}_{n+1}$ are unknown then

$$E(\vec{u}_{n+1}|\vec{o}_{n+1},\vec{y}_n) = \vec{y}_{un} + W_{u\cdot}\Delta t_{n+1}(\vec{y}_n - \vec{\mu}_n) + \boldsymbol{\Sigma}_{uo}\boldsymbol{\Sigma}_{oo}^{-1}(\vec{o}_{n+1} - \vec{y}_{on} - W_{o\cdot}\Delta t_{n+1}(\vec{y}_n - \vec{\mu}_n)). \tag{S3}$$

We used the simplest approach as an initial imputation (e.g. carry forward/back). We then imputed $\vec{y}_1$ first using equation (S1) then each subsequent timepoint using equation (S3).

We compared the imputation quality and found that the model mean was both straightforward and effective, and therefore elected to use it for both SLAM datasets and Paquid. We initialized imputation with carry forward/back: that is, forward carrying previous values until the last timepoint was reached then backwards carrying to fill any values still missing. In rare cases a few data points were missing after imputation, these were simply ignored (we used all available case data). The ELSA dataset was sensitive to the model mean — perhaps due to the limited number of data points — hence we used a single imputation which combined first imputing the individual-specific variable mean followed by the conditional mean, assuming multivariate Gaussian statistics at each timepoint. Note that since we elected to use bootstrapping, we imputed each bootstrap replicate and then averaged to get an estimate for each missing value along with a standard error.

The final imputation was assessed for quality, Figure S2 — and looked reasonable. When inspecting imputation quality we are looking for the same age-dependent pattern for both the imputed and observed values, both in terms of mean and dispersion. Informative censorship is possible, so for variables with survival effects we can expect that missing values should be at higher risk because they include individuals whom were censored due to poor health (or death). Risk can be inferred by the direction of drift with respect to age: data points which look 'older' are likely higher risk. Hence imputed values may look a little 'older' than observed values.

S4.1 Informative censorship

Is it better to impute dropped individuals (dead, censored) or not? Dropped individuals may have abnormal biomarker values leading to their exclusion, i.e. informative censorship. There is "substantial" evidence that dropped individuals in longitudinal studies have worse health[9] and their health biomarkers will reflect this, leading to a potential survivorship bias. We used

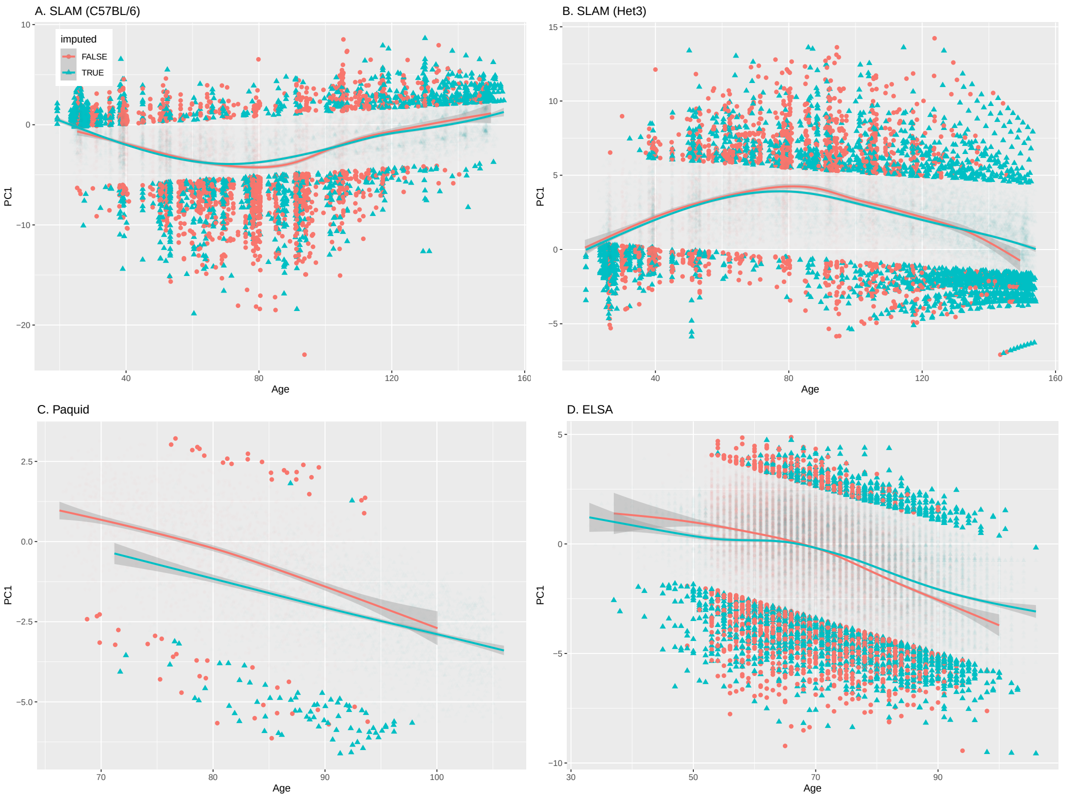

Figure S2. Final imputation quality check, visualized using principal component 1. **A.** C57BL/6 mice (SLAM). **B.** Het3 mice (SLAM). **C.** Paquid (human, dementia). **D.** ELSA (human). Imputed values appear to be reasonable for each dataset. Principal component analysis (PCA) was applied to each dataset in the entirety, flatted across timepoints. Good quality imputation (blue triangles) should show the same trend and dispersion as the observed data (red points). Censored individuals likely have worse health, so imputed values may look a little 'older' than the observed. Age-dependence is indicated by the solid lines with confidence intervals (cubic spline; `geom_smooth` with defaults[8]). Outlying points are highlighted ($l \pm 3$ where l is the ordinary linear regression model). Data points were labelled as imputed (blue triangles) if the preponderance of the rotation weights were missing: $\sum_{i=missing} |U_{i1}|/(\sum_j |U_{j1}|) > 0.5$; where $\boldsymbol{U}$ is the PCA rotation matrix.

simulated data to test for potential bias and observed that — if done well — imputing values for dropped individuals can reduce this bias.

We simulated data from our model equation (S4) using randomly generated parameters then imposed informative censorship. We simulated 100 times with 100 individuals in each simulation. Each simulation included 2 biomarkers. Parameters and biomarkers were draw from normal random variables. The diagonal of $\boldsymbol{W}$ was mean $-1/4$, the off-diagonals were mean 0 and the overall standard deviation was 0.1. The mean μ_0 was 0 and standard deviation was 0.1. No covariates were simulated. The noise was diagonal, $\boldsymbol{\Sigma} = 0.5\boldsymbol{I}$ (also used for instantiating the population). We censored using Gompertz statistics with proportional hazards for biomarker values[10] (shape: $\alpha = 0.1$, scale: $\lambda = 10^{-5}$). The proportional hazards coefficients were randomly sampled from a normal distribution with mean 1 and standard deviation 0.1, this ensured that large values of the biomarkers were preferentially censored.

The results of the simulation are shown in Figure S3 for various imputation strategies, with the horizontal dashed line indicating unbiased results. We observed that a significant bias existed in both the diagonal and off-diagonal elements of $\boldsymbol{W}$, which were systematically over-estimated if the data were not imputed. Conversely, if we used only the simple carry forward/back imputation, an even worse bias ensued in the opposing direction. If we used the model mean imputation, however, we reduced the bias in $\boldsymbol{W}$ to nearly 0 without significantly increasing bias in the other parameters. For this reason, we imputed all dropped individuals: censored and dead.

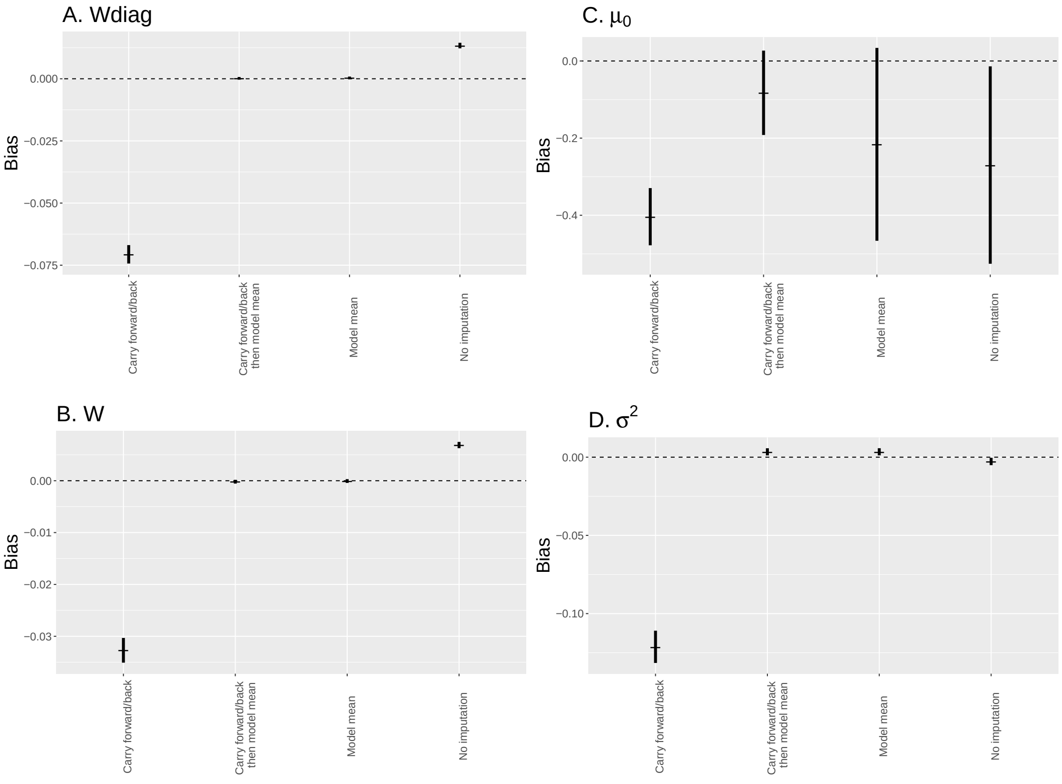

Figure S3. Imputation of dropped individuals can reduce bias. We simulated informative censorship and here compare estimates from different missing data handling strategies. Observe that both the diagonal elements of $\boldsymbol{W}$ (A.) and all elements of $\boldsymbol{W}$ (B.) were biased high when data were not imputed. However, if we imputed using the model mean, the bias was greatly reduced. For μ_0 (C.) we also reduced the bias with the combined imputation strategy, which was the strategy employed on the real data. Imputation did introduce a small bias in the noise estimate (D.). The bias was largest if we used only the carry forward/back method.

S5 Model selection

Our goal with model selection was two fold: (i) to find the optimal model(s) that best fit the data, and (ii) to test which model parameters were essential for fitting the data. This allows us to infer the existence of which model parameters are robustly supported by the data. Fit quality was measured using the root-mean squared error (RMSE) and mean absolute error (MAE). We used the 632 estimator for errors, which is a linear combination of 63.2% test error and 36.8% training error[11]. Test error was estimated via out-of-sample bootstrap replication, with 100 resamples. Each bootstrap selected a new dataset of the same size as the original by resampling individuals with replacement. The out-of-sample individuals are those whom were not selected. Estimation algorithms are reported in Section S6.

We compare model performance in Figure S4; we explain the model labels here. The general form of our model is ('full')

$$\begin{aligned}
\vec{y}_{n+1} &= \vec{y}_n + \boldsymbol{W}\Delta t_{n+1}(\vec{y}_n - \vec{\mu}_n) + \vec{\varepsilon}, \\
\vec{\varepsilon} &\sim \mathcal{N}(0, \Sigma|\Delta t|) \\
\vec{\mu}_n &\equiv \vec{\mu}_0 + \boldsymbol{\Lambda}\vec{x}_n + \vec{\mu}_{age} t_n,
\end{aligned} \tag{S4}$$

where t is the age. The error can be expressed in terms of the precision matrix,

$$\boldsymbol{Q} \equiv \boldsymbol{\Sigma}^{-1}. \tag{S5}$$

We considered both using $\boldsymbol{Q} = \boldsymbol{I}$, the identity matrix (default), and estimating $\boldsymbol{Q}$ from the data using the log-likelihood ('Q'). Transforming into natural variables — wherein $\boldsymbol{W}$ is diagonal — we have

$$z_{jn+1} = z_{jn} + \lambda_j \Delta t_{n+1}(z_{jn} - \tilde{\mu}_{jn}) + \tilde{\varepsilon}, \tag{S6}$$

where $\vec{z}_n \equiv \boldsymbol{P}^{-1}\vec{y}_n$, $\lambda_i \equiv P_{i\cdot}^{-1}\boldsymbol{W}P_{\cdot i}$, $\tilde{\vec{\mu}}_n \equiv \boldsymbol{P}^{-1}\mu_n$ and $\tilde{\vec{\varepsilon}} \equiv \boldsymbol{P}^{-1}\vec{\varepsilon}$.

We considered ('pca') the possibility that principal component analysis (PCA) could be used as a preprocessing step to decouple the biomarkers such that we could fit equation (S6), assuming independent noise between the z_j. Equation (S49) states that in the steady-state the principal components are equivalent to the eigenvectors of $\boldsymbol{W}$, this self-consistency motivates using PCA. Prior work has also suggested that principal components don't change much during the aging process[12].

We considered that μ_n may be time-dependent and may also depend on other covariates ('covs'), which is discussed in Section S2.

We considered simpler, nested forms of equation (S4). Recall that the data were standard-deviation-scaled and mean-centered by the baseline value, which justifies some of the simplifications. Simplified forms allowed us to test whether $\boldsymbol{W}$ and $\vec{\mu}$ were necessary to fit the data. Removing these parameters leads to special cases of the model. The simplest model for the data is to simply carry forward the previous value. If recovery is small, $W\Delta t \to 0$ then we have ('carry'),

$$\vec{y}_{n+1} = \vec{y}_n + \tilde{\varepsilon} \tag{S7}$$

which corresponds to carrying the previous value forward ($\langle\vec{y}_{n+1}\rangle = \langle\vec{y}_n\rangle$). This model does not require any parameters to make predictions; it was used for the initial imputation of the Paquid and SLAM datasets (Section S4).

If recovery is complete between each timepoint then $\boldsymbol{W}\Delta t \to -\boldsymbol{I}$ and we instead have the second simplest model ('fast'),

$$\vec{y}_{n+1} = \vec{\mu}_n + \vec{\varepsilon} \tag{S8}$$

which corresponds to biomarkers being randomly distributed about some mean value which depends on covariates ($\langle\vec{y}_{n+1}\rangle = \langle\vec{\mu}_n\rangle$). Alternatively, we could have $\vec{\mu}_n \equiv 0$ in which case we have ('noallo'),

$$\vec{y}_{n+1} = \vec{y}_n + W\Delta t_{n+1}\vec{y}_n + \vec{\varepsilon} \tag{S9}$$

which we refer to as the no allostasis model (it also implicitly sets homeostatic equilibrium to 0). In 1-dimension, equation (S9) is simple exponential growth/decay in the mean (for small Δt).

While nonlinear behaviour can be captured by our model (Section S8), we also directly investigated nonlinear behaviour by including a quadratic term ('quad'),

$$z_{jn+1} = z_{jn} + \lambda_j\Delta t_{n+1}(z_{jn} - \tilde{\mu}_{jn}) + \gamma\Delta t_{n+1}^2 z_{jn}^2 + \tilde{\varepsilon}. \tag{S10}$$

We only considered a quadratic term for the diagonal model, equation (S6) with PCA preprocessing.

We compare model performance in Figure S4. We found that the fast model, equation (S8), fit very poorly, having error so large that it did not fit in the plot region. Within the plot region, the carry-forward model performed the worst, equation (S7) ('carry'). Note the implication: biomarker recovery towards equilibrium is much closer to none ('carry') than complete ('fast'). Next worse was excluding μ, equation (S9) ('noallo'). The remaining models typically performed similarly-well. The SLAM datasets both saw a noteworthy improvement in fit when age was included as a covariate (in μ_n). We observed no improvement with inclusion of a quadratic term, equation (S10) ('quad'). Finally, and importantly, we found that a diagonal fit on principal components (PCs) yielded equivalent performance to the full model. This permitted a greatly simplified methodology since we were able estimate using weighted linear regression (Section S6.1).

S6 Estimation

We provide useful results for fitting equation (S4) and its simplified form, equation (S6). The latter can be solved using weighted linear regression.

S6.1 (Weighted) Linear Regression

If the noise term is diagonal then the equations decouple and we have a set of linear equations which can be independently solved using linear regression. In the present study we used PCA (principal component analysis) as a preprocessing step prior to fitting a diagonal model. That is, we assumed the PCs do not interact with each other. We can rewrite equation (S6) as

$$\begin{aligned} z_{ijn+1} - z_{ijn} &= \lambda_j(\Delta t_{in+1} z_{ijn}) + \vec{\beta}_j^{\,T}(\Delta t_{in+1}\vec{x}_{in}) + \varepsilon_{ij}, \quad \text{where} \\ \beta_{j0} &= \lambda_j\mu_0, \\ \beta_{jage} &= \lambda_j\mu_{jage}, \\ \beta_{jk} &= \lambda_j\Lambda_{k\cdot}, \quad \text{and} \\ \varepsilon_{ij} &\sim \mathcal{N}(0, \sigma_j^2|\Delta t_{in+1}|). \end{aligned} \tag{S11}$$

This is a weighted linear regression problem[13] where the predictors are $\Delta t_{in+1}z_{ijn}$, and $\Delta t_{in+1}x_{ijn}$; the weights are $|\Delta t_{in+1}|^{-1}$. During model selection, Section S5, we found that equation (S11) fit the data as well as the full model, equation (S4).

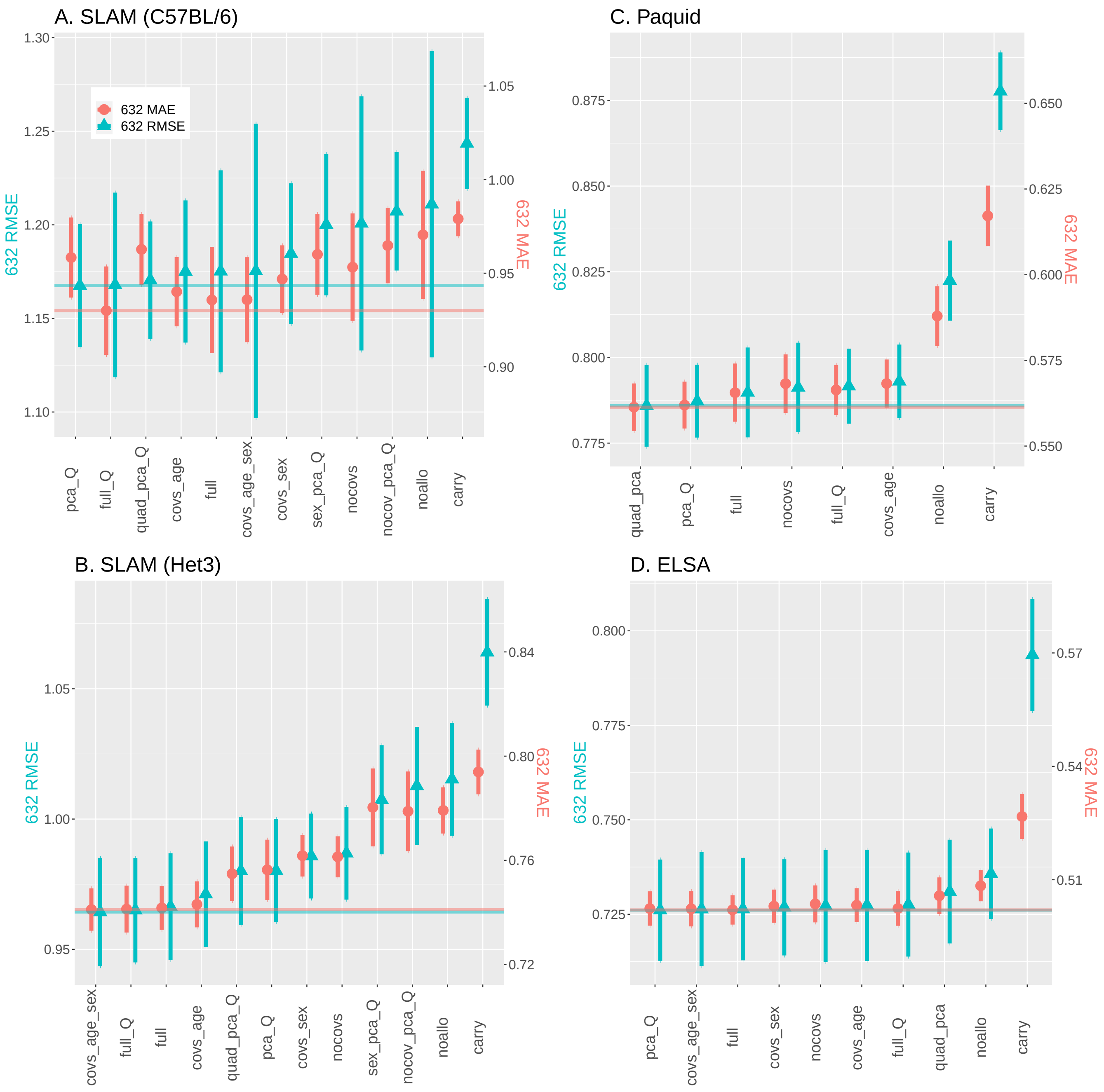

Figure S4. Model selection. **A.** C57BL/6 mice (SLAM). **B.** Het3 mice (SLAM). **C.** Paquid (human, dementia). **D.** ELSA (human). Lower error is better. y-axis is 632-RMSE on left and 632-MAE on right. Horizontal lines indicate the best performing model. We are looking for the simplest model that consistently hits those lines across datasets. We considered models significantly worse if they do not have an error interval overlapping this line; prioritizing RMSE. Models: carry: equation (S7); fast: equation (S8); noallo: equation (S9); quad: equation (S10); full: equation (S4). Additional parameters: pca: equation (S6) with PCA preprocessing and diagonal noise; Q: the noise was estimated; covs: prefix, after which included covariates are listed; nocovs: no covariates were used. For example, `sex_pca_Q` included sex as a covariate (`sex`), used PCA as a preprocessing step and assumed diagonal $\boldsymbol{W}$ and $\boldsymbol{Q}$, and fit equation (S6) (`pca`), and estimated Q from the data (`Q`). The fast model, equation (S8), performed much worse for all datasets (points above plot region), 632-RMSE: 0.91(2) (Paquid), 0.92(1) (ELSA), 2.03(6) (SLAM C57) and 2.21(7) (SLAM HET3); 632-MAE: 0.68(1) (Paquid), 0.702(5) (ELSA), 1.32(3) (SLAM C57) and 1.47(3) (SLAM HET3).

S6.2 Maximum likelihood estimators (MLEs)

We derive the MLEs for equation (S4) in full generality. For convenience define

$$\hat{y}_{ibn+1} \equiv y_{ibn} + \Delta t_{in+1} \sum_k W_{bk}(y_{ikn} - \mu_{ikn}) = y_{ibn} + \Delta t_{in+1} \sum_k W_{bk}(y_{ikn} - \sum_j \Lambda_{kj} x_{ijn}) \tag{S12}$$

where we have p variables, N individuals and $T+1$ timepoints. We index the N individuals with i and the T timepoint-pairs with n. For convenience we drop μ_0 and define the equivalent $\vec{\mu}_{in} \equiv \boldsymbol{\Lambda}\vec{x}_{in}$; where we use $x_{i0n} \equiv 1$ to recover μ_0. Estimators are denoted with a hat e.g. $\hat{\boldsymbol{W}}$ estimates $\boldsymbol{W}$.

The log-likelihood is,

$$\begin{aligned} l &= -\frac{1}{2}\sum_{i,n} \ln\left(\det\left|2\pi \boldsymbol{Q}^{-1}|\Delta t_{in+1}|\right|\right) - \frac{1}{2}\sum_{i,n}(\vec{y}_{in+1} - \hat{\vec{y}}_{in+1})^T \frac{\boldsymbol{Q}}{|\Delta t_{in+1}|}(\vec{y}_{in+1} - \hat{\vec{y}}_{in+1}) \\ &= \frac{1}{2} NT \ln\left(\det|\boldsymbol{Q}|\right) - \frac{p}{2}\sum_{i,n}\ln\left(2\pi|\Delta t_{in+1}|\right) - \frac{1}{2}\sum_{i,n}(\vec{y}_{in+1} - \hat{\vec{y}}_{in+1})^T \frac{\boldsymbol{Q}}{|\Delta t_{in+1}|}(\vec{y}_{in+1} - \hat{\vec{y}}_{in+1}). \end{aligned} \tag{S13}$$

We derive analytical forms for the MLEs as well as providing derivatives for gradient-based optimization algorithms. We also report the curvature since this is used to estimate the asymptotic error via the inverse Fisher matrix[14]. We found that the asymptotic errors are well-calibrated for $\boldsymbol{W}$, but tend to be too small for $\vec{\mu}_n$ (Section S7). In the present study, we report bootstrap errors.

Note that we find it useful to express the estimators in terms of the uncentered (cross)covariance,

$$\begin{aligned} \mathrm{Cov}_2(\vec{x}_{in}) &\equiv \langle \vec{x}\vec{x}^T \rangle_{i,n}, \text{ and} \\ \mathrm{Cov}_2(\vec{x}_{in}, \vec{y}_{in}) &\equiv \langle \vec{x}\vec{y}^T \rangle_{i,n} \end{aligned} \tag{S14}$$

where the expectation value is taken over individuals, i, and timepoints, n. In general, $\langle f(x_{in}) \rangle_{i,n}$ denotes the average of $f(x_{in})$ over individuals, i, and timepoints n.

We start by considering $\boldsymbol{W}$. The derivatives are

$$\begin{aligned} \frac{\partial l}{\partial W_{\alpha\beta}} &= \sum_{i,n,a} \mathrm{sign}(\Delta t_{in+1}) Q_{a\alpha}(y_{ian+1} - y_{ian} - \Delta t_{in+1}\sum_k W_{ak}(y_{ikn} - \mu_{ikn}))(y_{i\beta n} - \mu_{i\beta n}) \\ \nabla_W l &= \sum_{i,n} \mathrm{sign}(\Delta t_{in+1}) \boldsymbol{Q}(\vec{y}_{in+1} - \vec{y}_{in} - \Delta t_{in+1}\boldsymbol{W}(\vec{y}_{in} - \vec{\mu}_{in}))(\vec{y}_{in} - \vec{\mu}_{in})^T \end{aligned} \tag{S15}$$

where ∇_W denotes the gradient with respect to (vectorized) vec($\boldsymbol{W}$). The MLE is thus

$$\begin{aligned} \hat{\boldsymbol{W}}\langle|\Delta t_{in+1}|(\vec{y}_{in} - \vec{\mu}_{in})(\vec{y}_{in} - \vec{\mu}_{in})^T\rangle_{i,n} &= \langle \mathrm{sign}(\Delta t_{in+1})(\vec{y}_{in+1} - \vec{y}_{in})(\vec{y}_{in} - \vec{\mu}_{in})^T\rangle_{i,n} \\ \mathrm{Cov}_2(\sqrt{|\Delta t_{in+1}|}(\vec{y}_{in} - \vec{\mu}_{in}))\hat{\boldsymbol{W}}^T &= \mathrm{Cov}_2(\mathrm{sign}(\Delta t_{in+1})(\vec{y}_{in} - \vec{\mu}_{in}), \vec{y}_{in+1} - \vec{y}_{in}). \end{aligned} \tag{S16}$$

The latter equation is useful for linear algebra software packages. Alternatively, we can invert the uncentered covariance $\langle|\Delta t_{in+1}|(\vec{y}_{in} - \vec{\mu}_{in})(\vec{y}_{in} - \vec{\mu}_{in})^T\rangle_{i,n}$ which yields equation (12).

The curvature of $\boldsymbol{W}$ is

$$\frac{\partial^2 l}{\partial W_{\gamma\delta} W_{\alpha\beta}} = -NT Q_{\gamma\alpha}\langle|\Delta t_{in+1}|(y_{i\beta n} - \mu_{i\beta n})(y_{i\delta n} - \mu_{i\delta n})\rangle_{i,n} \tag{S17}$$

the Fisher information is the negative of this. The covariance of the MLE is given by the inverse Fisher information,

$$I^{-1}_{\alpha\beta\gamma\delta} = \frac{1}{NT} Q^{-1}_{\alpha\gamma}\langle|\Delta t_{in+1}|(\vec{y}_{in} - \vec{\mu}_{in})(\vec{y}_{in} - \vec{\mu}_{in})\rangle^{-1}_{\beta\delta} \tag{S18}$$

the standard errors are the square-roots of the diagonal elements,

$$\delta W^2_{\alpha\beta} = \frac{Q^{-1}_{\alpha\alpha}}{NT}\langle|\Delta t_{in+1}|(\vec{y}_{in} - \vec{\mu}_{in})(\vec{y}_{in} - \vec{\mu}_{in})\rangle^{-1}_{\beta\beta}. \tag{S19}$$

Next we consider $\vec{\mu}_n$. We condense all relevant parameters into $\mathbf{\Lambda}$ which has MLE,

$$\begin{aligned}\frac{\partial l}{\partial \Lambda_{\alpha\beta}} &= -\sum_{i,n,a,b} \frac{Q_{ab}}{|\Delta t_{in+1}|}(y_{ian+1}-\hat{y}_{ian+1})(\Delta t_{in+1} W_{b\alpha} x_{i\beta n}) \\ &= -NTW^T_{\alpha\cdot}\boldsymbol{Q}\langle(\vec{y}_{in+1}-\vec{y}_n)x_{i\beta n}\mathrm{sign}(\Delta t_{in+1})\rangle + NTW^T_{\alpha\cdot}\boldsymbol{QW}\langle|\Delta t_{in+1}|\vec{y}_{in}x_{i\beta n}\rangle \\ &\quad - W^T_{\alpha\cdot}\boldsymbol{QW\Lambda}\langle|\Delta t_{in+1}|\vec{x}_{in}x_{i\beta n}\rangle \\ \implies \frac{1}{NT}\nabla_\Lambda l &= -\boldsymbol{W}^T\boldsymbol{Q}\langle(\vec{y}_{in+1}-\vec{y}_n)\vec{x}^T_{in}\mathrm{sign}(\Delta t_{in+1})\rangle + \boldsymbol{W}^T\boldsymbol{QW}\langle|\Delta t_{in+1}|\vec{y}_{in}\vec{x}^T_{in}\rangle \\ &\quad - \boldsymbol{W}^T\boldsymbol{QW\Lambda}\langle|\Delta t_{in+1}|\vec{x}_{in}\vec{x}^T_{in}\rangle \end{aligned} \tag{S20}$$

This implies

$$\boldsymbol{W}^T\boldsymbol{QW}\hat{\mathbf{\Lambda}}\langle|\Delta t_{in+1}|\vec{x}_{in}\vec{x}^T_{in}\rangle = \boldsymbol{W}^T\boldsymbol{QW}\langle|\Delta t_{in+1}|\vec{y}_{in}\vec{x}^T_{in}\rangle - \boldsymbol{W}^T\boldsymbol{Q}\langle\mathrm{sign}(\Delta t_{in+1})(\vec{y}_{in+1}-\vec{y}_{in})\vec{x}^T_{in}\rangle \tag{S21}$$

which we can write

$$\begin{aligned}\boldsymbol{W}^T\boldsymbol{QW}\hat{\mathbf{\Lambda}} &= \boldsymbol{W}^T\boldsymbol{QW}\mathrm{Cov}_2(|\Delta t_{in+1}|\vec{y}_{in},\vec{x}_{in})\big(\mathrm{Cov}_2(\sqrt{|\Delta t_{in+1}|}\vec{x}_{in})^{-1}\big) \\ &\quad - \boldsymbol{W}^T\boldsymbol{Q}\mathrm{Cov}_2(\mathrm{sign}(\Delta t_{in+1})(\vec{y}_{in+1}-\vec{y}_{in}),\vec{x}_{in})\big(\mathrm{Cov}_2(\sqrt{|\Delta t_{in+1}|}\vec{x}_{in})^{-1}\big).\end{aligned} \tag{S22}$$

We estimate from the general form, but note that equation (S22) can be greatly simplified when $\boldsymbol{W}$ is invertible, which is expected because it empirically has strong diagonal elements. For invertible $\boldsymbol{W}$ we get equation (11).

The curvature is

$$\frac{\partial^2 l}{\partial \Lambda_{\gamma\delta}\partial \Lambda_{\alpha\beta}} = -(\boldsymbol{W}^T\boldsymbol{QW})_{\alpha\gamma}TN\langle|\Delta t_{in+1}|\vec{x}_{in}\vec{x}^T_{in}\rangle_{\beta\delta} \tag{S23}$$

where the expectation is over times and individuals. The Fisher information is used to estimate the asymptotic error,

$$I^{-1}_{\alpha\beta\gamma\delta} = \frac{1}{NT}(\boldsymbol{W}^T\boldsymbol{QW})^{-1}_{\alpha\gamma}\langle|\Delta t_{in+1}|\vec{x}_{in}\vec{x}^T_{in}\rangle^{-1}_{\beta\delta} \tag{S24}$$

the fit error is the square-root of the diagonal,

$$(\delta\Lambda_{\alpha\beta})^2 = \frac{1}{NT}(\boldsymbol{W}^T\boldsymbol{QW})^{-1}_{\alpha\alpha}\langle|\Delta t_{in+1}|\vec{x}_{in}\vec{x}^T_{in}\rangle^{-1}_{\beta\beta}. \tag{S25}$$

Finally, observe the equilibrium case where $\langle\vec{y}_{n+1}\rangle = \langle\vec{y}_n\rangle = \vec{\mu}_n$ and $\mathrm{Cor}(\vec{y}_{n+1}-\vec{y}_n,\vec{x}) = \mathrm{Cor}(\vec{y}_n-\vec{\mu}_n,\vec{x}) = 0$ (i.e. the fluctuations are random) then equation (S20) becomes

$$\frac{1}{NT}\nabla_{\Lambda_{eq}} l = \boldsymbol{W}^T\boldsymbol{QW}\langle|\Delta t_{in+1}|\vec{y}_{in}\vec{x}^T_{in}\rangle - \boldsymbol{W}^T\boldsymbol{QW\Lambda}\langle|\Delta t_{in+1}|\vec{x}_{in}\vec{x}^T_{in}\rangle \tag{S26}$$

and we have

$$\begin{aligned}\hat{\mathbf{\Lambda}}_{eq} &= \Big\langle|\Delta t_{in+1}|\vec{y}_{in+1}\vec{x}^T_{in}\Big\rangle\Big(\Big\langle|\Delta t_{in+1}|\vec{x}_{in}\vec{x}^T_{in}\Big\rangle\Big)^{-1}, \\ &= \mathrm{Cov}_2(|\Delta t_{in+1}|\vec{y}_{in+1},\vec{x}_{in})\big(\mathrm{Cov}_2(\sqrt{|\Delta t_{in+1}|}\vec{x}_{in})^{-1}\big).\end{aligned} \tag{S27}$$

equation (S27) is useful for an initial $\mathbf{\Lambda}$ estimate as it does not depend on $\boldsymbol{W}$.

S6.3 Noise estimator

We used a simple estimator for the noise, $\mathbf{\Sigma}$. For our model, equation (S4), a simple estimator is derived by observing

$$\vec{y}_{n+1} - \langle\vec{y}_{n+1}\rangle = \vec{\varepsilon} \tag{S28}$$

which implies that

$$\langle(\vec{y}_{n+1}-\langle\vec{y}_{n+1}\rangle)(\vec{y}_{n+1}-\langle\vec{y}_{n+1}\rangle)^T\rangle = \langle\vec{\varepsilon}\vec{\varepsilon}^T\rangle = \mathbf{\Sigma}|\Delta t| \tag{S29}$$

we conclude that

$$\hat{\mathbf{\Sigma}} = \Big\langle\frac{1}{|\Delta t_{in+1}|}(\vec{y}_{in+1}-\langle\vec{y}_{in+1}\rangle)(\vec{y}_{in+1}-\langle\vec{y}_{in+1}\rangle)^T\Big\rangle_{i,n}. \tag{S30}$$

Where the expectation must be taken over individuals, i, and timepoints, n. Note that $\vec{y}_{n+1}-\langle\vec{y}_{n+1}\rangle$ is the model residual, which is easily computed after the model has been fit.

S6.4 Iterative estimation

We found that a simple iterative scheme of alternating estimators from Section S6.2 was able to correctly recovery true parameter values in a simulation study, Section S7. The scheme proceeds according to Algorithm S1 (see below). We defaulted to numIter $= 5$ iterations. Note that in the special, diagonal case, of equation (S6) we simultaneously estimated $\boldsymbol{\Lambda}$ and $\boldsymbol{W}$ using weighted linear regression, Section S6.1 ("PCA" case).

While we did estimate the asymptotic error, we found that the bootstrapped error had lower bias and hence we only report the latter (see Section S7). To estimate the errors in parameters and prediction we bootstrapped Algorithm S1 and took the standard deviation as the error estimate (100 resamples).

Algorithm S1 Iterative estimator

---

if imputeFirst **then**
    Impute missing $\vec{y}$ using simple algorithm (e.g. carry forward/back).
end if
if doPCA **then**
    Estimate PCA rotation, $\boldsymbol{U}$, on first timepoint, $\vec{y}_1$, then apply to all timepoints.
end if
Estimate $\boldsymbol{\Lambda}$ using equation (S27) which assumes $\langle \vec{y}_{n+1} \rangle = \langle \vec{y}_n \rangle = \vec{\mu}_n$ and $\mathrm{Cor}(\vec{y}_n - \vec{\mu}_n, \vec{x}) = 0$.
Estimate $\boldsymbol{W}$ using equation (S16).
for i in 1 to numIter **do**
    if estimateNoise **then**
        Estimate $\boldsymbol{\Sigma}$ using equation (S30) and $\boldsymbol{Q} = \boldsymbol{\Sigma}^{-1}$.
    end if
    if imputeMean **then**
        if doPCA **then**
            Transform model parameters into observed space using $\boldsymbol{U}^{-1} = \boldsymbol{U}^T$.
        end if
        Impute $\vec{y}_1$ with the model mean using equation (S1).
        for n in 2 to numTimes **do**
            Impute $\vec{y}_n$ with the model mean using equation (S3).
        end for
    end if
    Estimate $\boldsymbol{\Lambda}$ using equation (S22).
    Estimate $\boldsymbol{W}$ using equation (S16).
end for
if doPCA **then**
    Transform imputed values and parameters into observed space using $\boldsymbol{U}^{-1} = \boldsymbol{U}^T$.
end if
Estimate asymptotic errors.
Return $\boldsymbol{W}$, $\boldsymbol{\Lambda}$, $\boldsymbol{\Sigma}$ and imputed values, $\vec{y}_{imp}$.

Where imputeFirst, doPCA, estimateNoise and imputeMean are Boolean user settings. numTimes is the number of observation timepoints in the dataset, $T+1$.

S7 Validation

We used synthetic (simulated) data to validate: (i) Algorithm S1, (ii) the parameter errorbars, and (iii) the prediction error estimator (RMSE). We used synthetic data based on the SLAM C57/BL6 dataset for validation. We fit the full model equation (S4) to the dataset: all 6 predictors and 2 covariates (sex and age), as well as estimating the noise. We used the fit parameters to generate new data then tested to see if Algorithm S1 recovered the true parameters and errors. Algorithm S1 was bootstrapped 100 times, the prediction error was estimated using both in-sample (train) and out-of-sample (test). We simulated 1000 times for each synthetic dataset size: 10, 50, 100, 500 and 1000 individuals. Each dataset had 22 timepoints.

We confirmed that Algorithm S1 is able to accurately reproduce true model parameters. In Figure S5 we plot the parameter estimates versus the ground truth values. We see that the algorithm is accurate for $N \geq 50$. We see a bias-low for the diagonal elements of $\boldsymbol{W}$.

S7.1 Parameter error

Here we test the calibration of our parameter errorbars. We compare both the bootstrap and asymptotic error estimates to the ground truth. Bootstrap errors were estimated using the standard deviation of bootstrap replicates. Asymptotic errors were estimated using the estimators in Section S6.2. As we will demonstrate in this section, the asymptotic errorbars can be too small, whereas the bootstrap errors appeared to be correctly calibrated. For this reason, we always used the bootstrap estimates in the main text. The asymptotic error estimates are much faster to compute and are presented for posterity.

In Figure S6 we present the coverage of both error estimators. The coverage is the fraction of times that the true parameter value fell within the estimated error interval. The nominal coverage is 68.3% for a normal random variable. In Figure S6A we present the coverage of the asymptotic error estimates and find that they are unsatisfactory for μ_{age} and μ_0 (errorbars were too small). These may be due to strong correlations between the two parameters, for example the parameters for body weight correlated across simulations at $\mathrm{cor}(\mu_{age}, \mu_0) = -0.886$, which could make the asymptotic errors inaccurate. In Figure S6B we observe that all of the bootstrap error parameter coverages were close to the nominal rate (dashed line), and were symmetrically distributed above and below. This indicates that our bootstrap parameter errorbars were properly calibrated.

S7.2 Prediction error

Our primary measure of prediction error was the root-mean-squared error (RMSE). It is important that our measure is properly calibrated such that it estimates the correct RMSE, i.e. in a simulation study where the true error is known. We compare three RMSE estimators to the ground truth: (i) the testing error, which is the out-of-sample bootstrap error, (ii) the training error, which is the in-sample bootstrap error, and (iii) the 632 error which is a linear combination of 63.2% testing error and 36.8% training error[11]. The ground truth error is the error of the sample given the correct parameter values: this is the error of a single sample, not the distribution of possible values. The average ground truth error should be an unbiased estimate of the true, distribution error. In Figure S7A we demonstrate that the 632 error is close to the ground truth error. In Figure S7B we present the coverage of each estimator and find they are all close to the nominal rate. We conclude that the 632 error is a satisfactory estimator of the true model error.

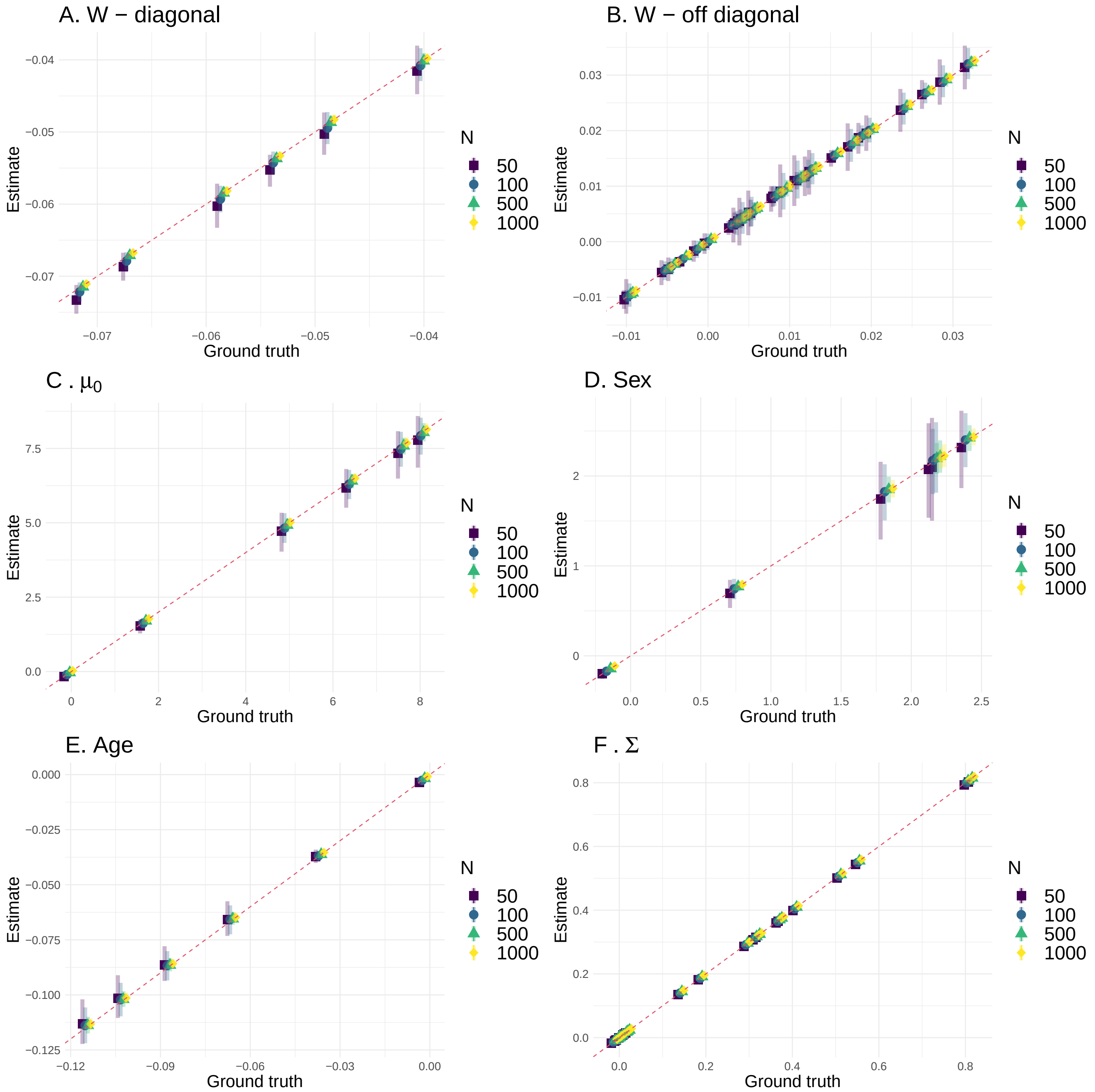

Figure S5. Algorithm S1 validation. For the indicated parameters in each measurement (A.-F.), the estimated value is plotted against the ground truth value for a variety of sample sizes (indicated by the legend). Points show mean; bands are the interquartile range (25th to 75th percentile). Bias is indicated by position of point relative to the red dashed line, $y = x$ (perfect estimator). Precision (and accuracy) are inferred by the dispersion (bands). As the number of individuals, N, is increased from 50 to 1000 we see the estimator becomes increasingly accurate and precise, with a small dispersion around the ground truth values for each parameter. Points are staggered for visualization. Note: $N = 10$ had large errors and hence was excluded for better visualization.

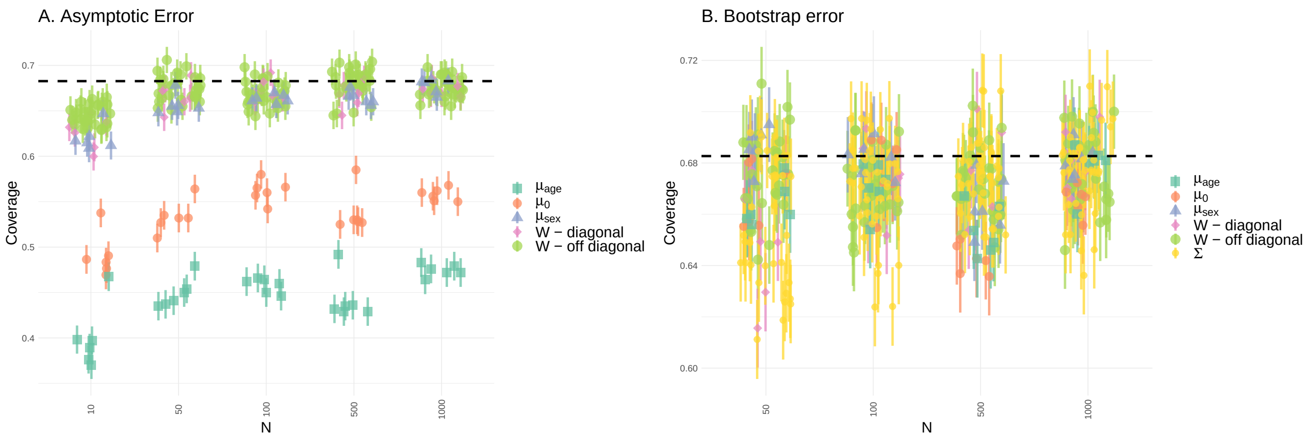

Figure S6. Parameter errorbar validation (coverage). Asymptotic errorbars can be too small, whereas bootstrap errorbars appear to be valid. **A.** Asymptotic error clearly has abnormally low coverage for μ_0 and μ_{age}, perhaps due to strong correlations between the two parameters. Asymptotic error estimates for the other parameters look good. **B.** bootstrap error coverage looks good: parameters are close to the nominal rate (dashed line) and are (mostly) symmetrically distributed above and below. Note the scale. Errorbars are standard error in the mean. x-axis not to scale.

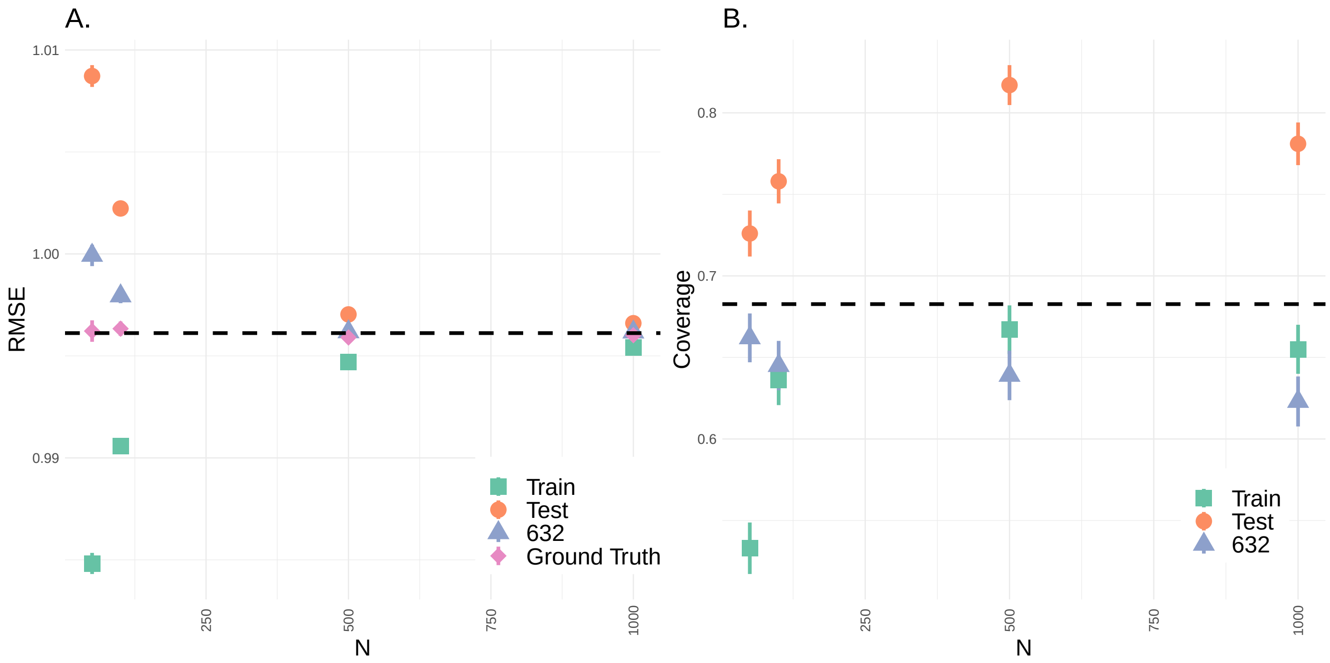

Figure S7. Bootstrap error calibration. 632 error is a satisfactory estimator of the true error. **A.** Test error (out-of-sample) was biased high, training error (in-sample) was biased low, whereas 632 error was nearly unbiased relative to the ground truth. **B.** The coverage of the train and 632 error were close to the nominal rate, 68.3% (dashed line). The test error clearly had abnormally high coverage, indicating the errorbars on the test error are too large. Note: the true (stochastic) error is difficult to precisely estimate due to non-uniform sampling, so we used the average ground truth to estimate the true error. Errorbars are standard error.

S8 Math

In this section we include supporting information for the model moments along with mappings to related approaches, i.e. other researcher's models.

S8.1 Ordinary differential equation

We consider only 1-dimension since we found that we could transform our multivariate biomarkers into a set of decoupled, 1-dimensional equations using the $\boldsymbol{W}$-diagonalizing matrix, $\boldsymbol{P}$. That is,

$$\vec{z} \equiv \boldsymbol{P}^{-1}\vec{y} \tag{S31}$$

decouples the z_j into a set of independent 1-dimensional equations. As needed, we can map back into $\vec{y}$ — which we do in Section S8.5.

In the limit of small Δt our 1-dimensional model equation (S6) becomes a modified Ornstein-Uhlenbeck process as follows,

$$\lim_{\Delta t \to 0} z_{jn+1} = z_{jn} + \lambda_j (z_{jn} - \tilde{\mu}_{jn}) dt + \lim_{\Delta t \to 0} \tilde{\varepsilon}, \tag{S32}$$

with

$$\tilde{\varepsilon} \sim \mathcal{N}(0, \tilde{\sigma}_j^2 |\Delta t|). \tag{S33}$$

A Wiener process, $d\xi$, has three criteria[15]: (i) independence, (ii) stationarity (statistics doesn't change over time), and (iii) $\mathcal{N}(0, |\Delta t|)$–distributed. These criteria are satisfied by $\tilde{\varepsilon}$ once we scale out $\tilde{\sigma}_j$. Substituting t for timepoint n and $t + dt$ for timepoint $n+1$ we have

$$z_j(t+dt) = z_j(t) + \lambda_j (z_j(t) - \tilde{\mu}_j(t)) dt + \tilde{\sigma}_j d\xi(t), \tag{S34}$$

which can be rewritten as

$$dz_j(t) = \lambda_j (z_j(t) - \tilde{\mu}_j(t)) dt + \tilde{\sigma}_j d\xi(t), \tag{S35}$$

which is an Ornstein-Uhlenbeck process with a non-constant equilibrium, $\tilde{\mu}_j(t)$, which depends on time through $\tilde{\mu}_{j,age} t$[15]. Note that equation (S35) holds for all $\tilde{\mu}_j(t)$ that can be Taylor expanded, since the nonlinear corrections go as $\mathcal{O}(\Delta t^2)$.

We rewrite equation (S35) in a stripped down form as

$$dz = \lambda (z - \mu(t)) dt + \sigma d\xi. \tag{S36}$$

The solution is then

$$z(t) = z_0 e^{\lambda t} - \lambda e^{\lambda t} \int_0^t \mu(s) e^{-\lambda s} ds + \sigma e^{\lambda t} \int_0^t e^{-\lambda s} d\xi(s). \tag{S37}$$

The integral is stochastic (Ito) and cannot be solved analytically, however its moments can be computed using two standard results[15]. The mean is

$$\langle \int_0^t f(s,\xi) d\xi \rangle = 0, \tag{S38}$$

and the two-point correlations are

$$\langle \int_0^t f(s,\xi) d\xi(s) \int_0^{t'} g(u,\xi) d\xi(u) \rangle = \int_0^{\min(t,t')} \langle f(s,\xi) g(s,\xi) \rangle ds. \tag{S39}$$

The statistics are Gaussian so all moments can be rewritten in terms of the mean and two-point correlations.

S8.1.1 Moments

Here we provide additional details supporting the math in Box 1.

Let

$$\mu(t) = \mu_0 + \mu_{age} t, \tag{S40}$$

that is, the only time-dependence is through the linear term $\mu_{age} t$ (μ_0 can still depend on covariates, but they can't vary in time).

Starting from equation (S37)

$$
\begin{aligned}
z(t) &= z_0 e^{\lambda t} - \lambda e^{\lambda t} \int_0^t \mu(s) e^{-\lambda s} ds + \sigma e^{\lambda t} \int_0^t e^{-\lambda s} d\xi(s) \\
&= z_0 e^{\lambda t} - \lambda e^{\lambda t} \left(-\frac{\mu_0}{\lambda} \left(e^{-\lambda t} - 1\right) + \frac{\mu_{age}}{\lambda^2} \left(e^{-\lambda t}(-\lambda t - 1) + 1\right) \right) + \sigma e^{\lambda t} \int_0^t e^{-\lambda s} d\xi(s).
\end{aligned} \tag{S41}
$$

The mean is easily computed as

$$
\begin{aligned}
\langle z(t) \rangle &= \langle z(0) \rangle e^{\lambda t} - \lambda e^{\lambda t} \left(-\frac{\mu_0}{\lambda} \left(e^{-\lambda t} - 1\right) + \frac{\mu_{age}}{\lambda^2} \left(e^{-\lambda t}(-\lambda t - 1) + 1\right) \right) \\
&= \langle z(0) \rangle e^{\lambda t} + (\mu_0 + \frac{\mu_{age}}{\lambda})(1 - e^{\lambda t}) + \mu_{age} t.
\end{aligned} \tag{S42}
$$

The auto-covariance doesn't depend on $\mu(t)$, it is simply,

$$
\begin{aligned}
\Big\langle (z(t+\tau) - \langle z(t+\tau) \rangle)(z(t) - \langle z(t) \rangle) \Big\rangle &= \sigma^2 e^{2\lambda t} e^{\lambda \tau} \Big\langle \int_0^{t+\tau} e^{-\lambda s} d\xi(s) \int_0^t e^{-\lambda s} d\xi(s) \Big\rangle \\
&= -\frac{\sigma^2}{2\lambda} \left(e^{\lambda |\tau|} - e^{2\lambda t} e^{\lambda \tau} \right).
\end{aligned} \tag{S43}
$$

The variance is the special case $\tau = 0$,

$$
\mathrm{Var}(z(t)) = -\frac{\sigma^2}{2\lambda} \left(1 - e^{2\lambda t} \right). \tag{S44}
$$

The mean and auto-covariance completely characterize Gaussian statistics, all other statistics can be calculated from them.

The above moments neglect the possibility that we may be unable to measure the system at $t = 0$. It is therefore useful to define the moments relative to a reference time, t_r. Doing some algebra we have

$$
\begin{aligned}
\langle z(t) \rangle &= \langle z(t_r) \rangle e^{\lambda (t - t_r)} + (\mu_0 + \frac{\mu_{age}}{\lambda} + \mu_{age} t_r)(1 - e^{\lambda (t - t_r)}) + \mu_{age}(t - t_r) \\
&= \langle z(t_r) \rangle e^{\lambda (t - t_r)} + (\mu(t_r) + \frac{\mu_{age}}{\lambda})(1 - e^{\lambda (t - t_r)}) + \mu_{age}(t - t_r)
\end{aligned} \tag{S45}
$$

for the mean, where $\mu(t_r) \equiv \mu_0 + \mu_{age} t_r$. Note that it is convenient to write

$$
\langle z(t) \rangle - \mu(t) = (\langle z(t_r) \rangle - \mu(t_r)) e^{\lambda (t - t_r)} + \frac{\mu_{age}}{\lambda}(1 - e^{\lambda (t - t_r)}). \tag{S46}
$$

For the variance we have

$$
\mathrm{Var}(z(t)) = \mathrm{Var}(z(t_r)) e^{2\lambda (t - t_r)} - \frac{\sigma^2}{2\lambda} \left(1 - e^{2\lambda (t - t_r)} \right). \tag{S47}
$$

Note that if we wait a long time, $t - t_r \gg 1/\lambda$, we reach steady-state values (so long as $\lambda < 0$). For example, the steady-state variance is

$$
\mathrm{Var}(z)_{ss} = -\frac{\sigma^2}{2\lambda}. \tag{S48}
$$

S8.2 Biomarker Principal Components

The biomarkers, $\vec{y}$, are connected to the natural variables, $\vec{z}$, by the transformation, $\boldsymbol{P}^{-1}$, equation (S31). $\boldsymbol{P}^{-1}$ is the (linear) diagonalizing transformation of $\boldsymbol{W}$. We can use this to calculate the steady-state principal components of $\vec{y}$,

$$
\begin{aligned}
\mathrm{Cov}(\vec{y}_{ss}, \vec{y}_{ss}) &= \langle (\vec{y}_{ss} - \langle \vec{y}_{ss} \rangle)(\vec{y}_{ss} - \langle \vec{y}_{ss} \rangle)^T \rangle \\
&= \boldsymbol{P} \langle (\vec{z}_{eq} - \langle \vec{z}_{ss} \rangle)(\vec{z}_{eq} - \langle \vec{z}_{ss} \rangle)^T \rangle \boldsymbol{P}^T \\
&= \boldsymbol{P} \mathrm{Cov}(\vec{z}_{ss}, \vec{z}_{ss}) \boldsymbol{P}^T.
\end{aligned} \tag{S49}
$$

If $\boldsymbol{P}$ is a rotation/orthogonal ($\boldsymbol{P}^{-1} = \boldsymbol{P}^T$) then, by definition[16], $\boldsymbol{P}$ is the diagonalizing transformation of $\mathrm{Cov}(\vec{y}_{ss}, \vec{y}_{ss})$, with eigenvalues equal to the steady-state variance of the z_j. Note: $\boldsymbol{P}$ is orthogonal if $\boldsymbol{W}$ is real and symmetric[16]. If we rank-order the $\mathrm{Var}(z_j)$ then we have exactly the principal components of $\vec{y}$.[12] Hence, in the steady-state the principal components are exactly the same as the natural variables, $\vec{z}$, sorted in order of decreasing variance, equation (S48).

S8.3 Small Timesteps, Δt

Our model, equation (S4), approximates an ordinary differential equation in the limit $|\lambda \Delta t| \ll 1$ (Sections S8.1 and S8.5). Sehl and Yates[17] found that most biomarkers decay linearly at a rate of $\lambda < 0.01$ year^{-1} with the fastest being about 0.03 year^{-1}. The frailty index — the average number of health deficits an individual has — accumulates at a similarly small rate of $0.025 - 0.04$ year^{-1}.[18] We observed typical rates in the range $0.025 - 0.05$ human-equivalent year^{-1} (Figure 2B), with sampling times Δt of 4 years for ELSA, 3 years for Paquid, 4.9 human-equivalent years for SLAM C57/BL6 and 3.6 human-equivalent years for SLAM Het3. This implies that we can expect $|\lambda \Delta t| \ll 1$ and therefore the small Δt approximation of equation (S4) is likely fine. This means our model should behave similarly to an ordinary differential equation.

S8.4 General dynamics

Linear and nonlinear dynamical models alike can be analysed for stability near an equilibrium position using the eigenvalues[19]. The system is linearized as

$$\frac{d}{dt}\vec{y} = \boldsymbol{W}\vec{y} + \vec{b}. \tag{S50}$$

The system is stable if and only if the real parts of the eigenvalues are always negative (positive recovery). Observe that the mean of our model equation (S4) can be written as

$$\begin{aligned} \frac{\langle \vec{y}_{n+1} - \vec{y}_n \rangle}{\langle \Delta t_{n+1} \rangle} &= \boldsymbol{W}\langle \vec{y}_n \rangle - \boldsymbol{W}\vec{\mu}_n \\ &= \boldsymbol{W}\langle \vec{y}_n \rangle + \vec{b} \end{aligned} \tag{S51}$$

for $\vec{b} \equiv -\boldsymbol{W}\vec{\mu}_n$. Hence for small Δt we have (approximately)

$$\frac{d}{dt}\langle \vec{y}(t) \rangle = \boldsymbol{W}\langle \vec{y}(t) \rangle + \vec{b} \tag{S52}$$

hence our approach probes the mean-stability of arbitrary linear or nonlinear dynamics.

S8.5 Stochastic process model (SPM) approximation

Our model can be used to analyse arbitrary dynamical systems near equilibrium, as discussed in Section S8.4. Here we show how a specific dynamical model — the stochastic process model (SPM) — is approximated by our model. Our model was motivated in part by earlier works which have shown that biomarker data can be modelled as a stochastic differential equation[20,21]. The earlier work by Yashin *et al.* proposed the SPM as a generic framework for longitudinal aging biomarker data[20] where an individual's collection of biomarkers, $\vec{y}$, evolves over time as

$$d\vec{y} = \boldsymbol{A}(t)(\vec{y} - \vec{\mu}(t))dt + \boldsymbol{B}(t)d\xi_t \tag{S53}$$

where $\vec{\mu}$ is the unknown equilibrium term ("functional state" of the organism), $\boldsymbol{A}$ is the interaction network and $d\xi_t$ is a Wiener noise term modified by the matrix $\boldsymbol{B}$. Subsequent work by Farrell *et al.* demonstrated that a deep neural network could be used to fit SPM and further that a time-independent linear interaction model was sufficient to describe the interaction network, $\boldsymbol{A}$, for ELSA data[21]. Our model, equation (S4), is the appropriate approximation for equation (S53) for small timesteps.

Proof: In Section S8.1 we showed that our 1-dimensional model is equivalent to a Wiener process in the limit of $\Delta t \to 0$. Consider the SPM with constant regulation matrix, $\boldsymbol{A}$, and linear functional state, μ,

$$d\vec{y} = \boldsymbol{A}(\vec{y} - \vec{\mu}(t))dt + \boldsymbol{B}d\xi \tag{S54}$$

Suppose $\boldsymbol{A}$ is diagonalizable then,

$$\begin{aligned} d\vec{y} &= \boldsymbol{P}\boldsymbol{D}\boldsymbol{P}^{-1}(\vec{y} - \vec{\mu})dt + \boldsymbol{B}d\xi, \\ \implies d(P^{-1}\vec{y}) &= \boldsymbol{D}(\boldsymbol{P}^{-1}(\vec{y} - \vec{\mu}))dt + \boldsymbol{P}^{-1}\boldsymbol{B}d\xi, \\ \implies d\vec{z} &= \boldsymbol{D}(\vec{z} - \tilde{\vec{\mu}})dt + \tilde{\boldsymbol{B}}d\xi \end{aligned} \tag{S55}$$

for the latent space, $\vec{z} \equiv \boldsymbol{P}^{-1}\vec{y}$. By inspection, the latent space obeys Ornstein-Uhlenbeck dynamics with $D_{jj} = \lambda_j$ and hence we can approximate each z_j,

$$\begin{aligned} & z_j(t + \Delta t) \approx z_j(t) + D_{jj}(z_j(t) - \tilde{\mu}_j)\Delta t + \tilde{\varepsilon}_j, \quad \text{where} \\ & \tilde{\vec{\varepsilon}} \sim \mathcal{N}(0, \tilde{\boldsymbol{B}}\tilde{\boldsymbol{B}}^T|\Delta t|) \end{aligned} \tag{S56}$$

which we can map into the observed space using $\boldsymbol{P}$ to get,

$$\boxed{\vec{y}(t+\Delta t) \approx \vec{y}(t) + \boldsymbol{A}(\vec{y}(t) - \vec{\mu})\Delta t + \vec{\varepsilon}} \tag{S57}$$

which is equation (S4) with $\boldsymbol{A} \equiv \boldsymbol{W}$. The transformed variance of $\tilde{\varepsilon}_i$ is,

$$\begin{aligned}\langle(\boldsymbol{P}\tilde{\vec{\varepsilon}})(\boldsymbol{P}\tilde{\vec{\varepsilon}})^T\rangle &= \boldsymbol{P}\langle\tilde{\vec{\varepsilon}}\tilde{\vec{\varepsilon}}^T\rangle\boldsymbol{P}^T \\ &= \boldsymbol{P}\langle\tilde{\boldsymbol{B}}\tilde{\boldsymbol{B}}^T\rangle\boldsymbol{P}^T|\Delta t| \\ &= \langle\boldsymbol{B}\boldsymbol{B}^T\rangle|\Delta t| \\ &\equiv \boldsymbol{\Sigma}|\Delta t|\end{aligned} \tag{S58}$$

QED.

S8.6 Mapping to Sehl and Yates

Sehl and Yates performed a meta-analysis of 469 biomarkers across cross-sectional and longitudinal aging studies and observed that the vast majority of biomarkers decay linearly with age[17]. In the present section we demonstrate that their linear model describes the steady-state dynamics of our model. In other words, the long-time (old-age) behaviour of our model is consistent with their observations.

In the steady-state our model equation (7) becomes linear in time,

$$\langle z_{jn}\rangle_{ss} = \mu_{0j}(\vec{x}) + \mu_{age,j}t_n - \frac{\mu_{age,j}}{|\lambda_j|} \tag{S59}$$

where we have included all time-independent covariates in $\mu_0(\vec{x})$ for convenience. The biomarkers have a one-to-one relationship with the natural variables through the orthogonal transformation $\boldsymbol{P}$ and thus evolve according to

$$\langle y_{ln}\rangle_{ss} = \sum_j P_{lj}\mu_{age,j}t_n + \sum_j P_{lj}\left(\mu_{0j}(\vec{x}) - \frac{\mu_{age,j}}{|\lambda_j|}\right). \tag{S60}$$

The Sehl and Yates model[17] is

$$\frac{y_{ln}}{y_{l,30}} = 1 - k_l(t_n - 30) \tag{S61}$$

for biomarker y_l with baseline value of $y_{l,30}$ at age 30; the age is in years and k_l is the rate in %-change per year. We can rewrite their model as

$$y_{ln} = -k_l y_{l,30}\mathrm{t}_n + (30k_l + 1)y_{l,30}, \tag{S62}$$

which is exactly equation (S60) with the substitutions

$$\begin{aligned}\sum_j P_{lj}\mu_{age,j} &\equiv -k_l y_{l,30}, \quad \text{and} \\ \sum_j P_{lj}\left(\mu_{0j} - \frac{\mu_{age,j}}{|\lambda_j|}\right) &\equiv (30k_l+1)y_{l,30},\end{aligned} \tag{S63}$$

which can be mapped into $\vec{z}$ using $\boldsymbol{P}^{-1}$:

$$\begin{aligned}\mu_{age,j} &\equiv -\sum_l P^{-1}_{jl}k_l y_{l,30}, \quad \text{and} \\ \mu_{0j} - \frac{\mu_{age,j}}{|\lambda_j|} &\equiv \sum_l P^{-1}_{jl}(30k_l+1)y_{l,30}.\end{aligned} \tag{S64}$$

Observe that $\boldsymbol{P}$ permits the drift of only a few $\vec{z}$ to map into many observed biomarkers, $\vec{y}$. Together with our observation that many more biomarkers drift than do natural variables, Figure S10, this implies that Sehl and Yates' observation that most biomarkers drift with age may be due to a only few underlying (allostatic) natural variables that are declining with age.

S9 Additional Results

We restricted the main text to only our key results. Here we provide additional information to support our conclusions.

We included covariates, $\vec{x}$, in the equilibrium position, $\mu(\vec{x})$, to reduce confounding effects and to test for the presence of allostasis (which depends on age). Here we tested each parameter for significance using the bootstrap parameter error estimates. The z-score for each covariate is reported in Figure S8; blue tiles are not significant, white and red are significant at $p \leq 0.05$. Most covariates were significant, particularly age for the human studies. In Section S5 we found that the effect of covariates on prediction was typically small. This means that the effects of covariates were reliably estimated (small p) but did not explain much variation (minor effect on RMSE).

Our model estimates an interaction network, $\boldsymbol{W}$, together with equilibrium positions. In the main text we presented the ELSA network with suppressed diagonal (Figure 2). The complete networks for each dataset are provided in Figure S9. The networks are all symmetrical because we used PCA as a preprocessing step. Relationships indicate how the y-axis variable will affect the x-axis variable during the next timestep.

Our model also estimates an equilibrium homeostatic position for each variable, μ. An important question is how strongly do variables adhere to homeostasis in the biomarkers, $\vec{y}$ versus the natural variables, $\vec{z}$? In the main text we presented the difference between the natural variable mean and the equilibrium position for each variable, $\langle z_j - \mu_j \rangle$. We reproduce that figure beside the observed biomarkers in Figure S10. In Figure S10B (and Figure 3A) we observed that the natural variables appear to be split into two groups: the majority group was close to μ, indicative of homeostasis, whereas the minority group was far from homeostasis. This latter group had a strong drift term, μ_{age}, which indicated that homeostasis was a moving target i.e. allostasis. In Figure S10A we show that the observed biomarkers were much more likely to be far from homeostasis than the natural variables (B), implying that the natural variables are able to condense the effects of age-related drift (allostasis) into a few variables (see also Section S8.6).

The natural variables appear to be efficient for representing age-related changes. What do the natural variables mean in terms of observable outcomes? In Figure S11 we report the correlations between the accumulating/drifting natural variables and biomarkers. In Figure S12 we report correlations with covariates. Together these give us an idea of what each natural variable represents and, by model implication, is controlling. For example, z_1 of Paquid is strongly correlated with the mental acuity scores: MMSE, BVRT and IST, implying it represents overall mental acuity. This may explain why z_1 was such a strong predictor of dementia (Figure S14). In this way the age-related decline of mental acuity can be represented by changes to just one variable, z_1, but also observed across several biomarkers, MMSE, BVRT and IST.

The linear map, $\boldsymbol{P}$, allows a few natural variables to cause several biomarkers to drift. The effects of allostasis on observed biomarkers via the primary risk natural variables are illustrated in Figure S13. The sign of each natural variable is arbitrary, due to idiosyncrasies in eigendecompositions[12]. The dominant survival dimension for the Het3 mouse data was z_2, which appears to capture a loss of body fat and muscle, and relative gain of fluid. The dominant z_2 dimension for the C57BL/6 was more specific to loss of fat (the z_1 signal for C57BL/6 was very similar to the z_2 signal for Het3, Figure S11). z_1 was the dominant dementia-free-survival dimension for Paquid, and captured a system-wide drop in mental acuity scores (MMSE, BVRT and IST), which likely captures cognitive decline associated with dementia. z_1 for ELSA appears to be related to frailty[12], having its effects spread across many variables, especially those related to disability (eye, hear, FI ADL and FI IADL), physical condition (grip strength and gait speed), and self-reported health (SRH); note that higher is better for physical condition variables and worse for the other variables (eye, hear, SRH, FI, etc). In all cases the effects are strongest in the natural variables, which is ensured by the orthogonality of $\boldsymbol{P}$. This means that the effects of the natural variable drift must be diluted across the observed biomarkers (e.g. Figure S10), potentially hiding them within healthy variation.

The drift rate of the natural variables, μ_{age}, was correlated with the risk of adverse outcome (Figure 4A). We named this phenomenon "mallostasis": the tendency of an aging system towards an ever-worsening equilibrium. Here we consider the role of confounding variables by constructing complete survival models for each natural variable and adverse outcome (mortality or dementia onset). We constructed a (time-dependent) survival model for each natural variable, with age, sex and the natural variable as predictors. We then recorded the Cox proportional hazards coefficients, which represent the (conditional) log–hazard ratios per unit increase for each natural variable. We observed that the Cox coefficients correlated with the drift rate, μ_{age}: Figure S14. This provides more robust support for mallostasis: that the steady-state behaviour of aging mice and humans is declining natural variables and commensurately declining health.

As an illustration of mallostasis, we consider a simple composite health measure, $b \equiv \vec{\mu}_{age}^T \vec{z}$. Figure S14 demonstrates that Cox coefficient is proportional to μ_{age} therefore b is proportional to the hazard. This is confirmed in Figure S15 which demonstrates that b for each dataset is a good predictor of survival (or dementia onset).

The natural variables are connected to survival via mallostasis, but how do they relate to the observed phenotype? That is, how do the changes in the natural variables with age affect the observed biomarkers? The total mean and variance are conserved between the biomarkers and the natural variables by Parseval's theorem. This means that natural variables with large means and variances will dominate the means and variances of the observed biomarkers, thus controlling the major changes we see. The

steady-state mean grows indefinitely proportional to μ_{age}, what about the variance? The equilibrium (steady-state) dispersion, equation (S48), for the natural variables are plotted in Figure S16. Smaller eigenvalues are associated with higher variance. Some dimensions (e.g. z_1 for the C57BL/6) can contain as much as 10x more variance than the next highest dimension. These dimensions will dominate the observed variance in the steady-state. The model predicts that these dimensions will eventually become the dominant principal components (PCs), equation (S49), implying they would dominate the observed phenotype in the steady-state. Hence what we observe will be dominated by natural variables with small λ and large μ_{age}, such as z_1 of ELSA, which appears to be closely related to frailty.

We used PCA (principal component analysis) as a preprocessing step, which allowed us to fit a diagonal model, equation S6. This simplified analysis and yielded equivalent performance to the full model (Section S5). Here we test the self-consistency of the approach. By using PCA, at each bootstrap the eigenvectors of $\boldsymbol{W}$ are principal components, possibly reordered (because we fit a diagonal model for $\boldsymbol{W}$). Averaging over multiple bootstrap replicates removes this equivalence — although in the steady-state the model predicts that the principal components and eigenvectors of $\boldsymbol{W}$ will coincide, equation (S49). Here we test the similarity of the PCA rotation and the eigenvector rotation: if they coincide then the principal components are eigenvectors. In Figure S17 we present the inner product between these matrices, which varies from -1 to 1, with ± 1 representing perfect similarity. We observed strong similarities between the transformations, indicating that the principal components and natural variables will be strongly correlated. This suggests that PCA may be a useful shortcut for approximating the eigenvectors of $\boldsymbol{W}$.

Finally, we include a survival summary for each dimension in terms of conditional Cox regression and the C-index in Figure S18. The values are identical to those used in Figures S14 (Cox coefficient) and 4 (C-index). This permits the reader to investigate the relative importance of each dimension. Comparing to the correlates of each dimension, Figure S11, one can infer potential mechanisms. For example, z_2 of C57BL/6 has a strong survival effect (low is bad) and shows increasing glucose and fat, which could indicate metabolic dysfunction, which C57BL/6 are prone to[22].

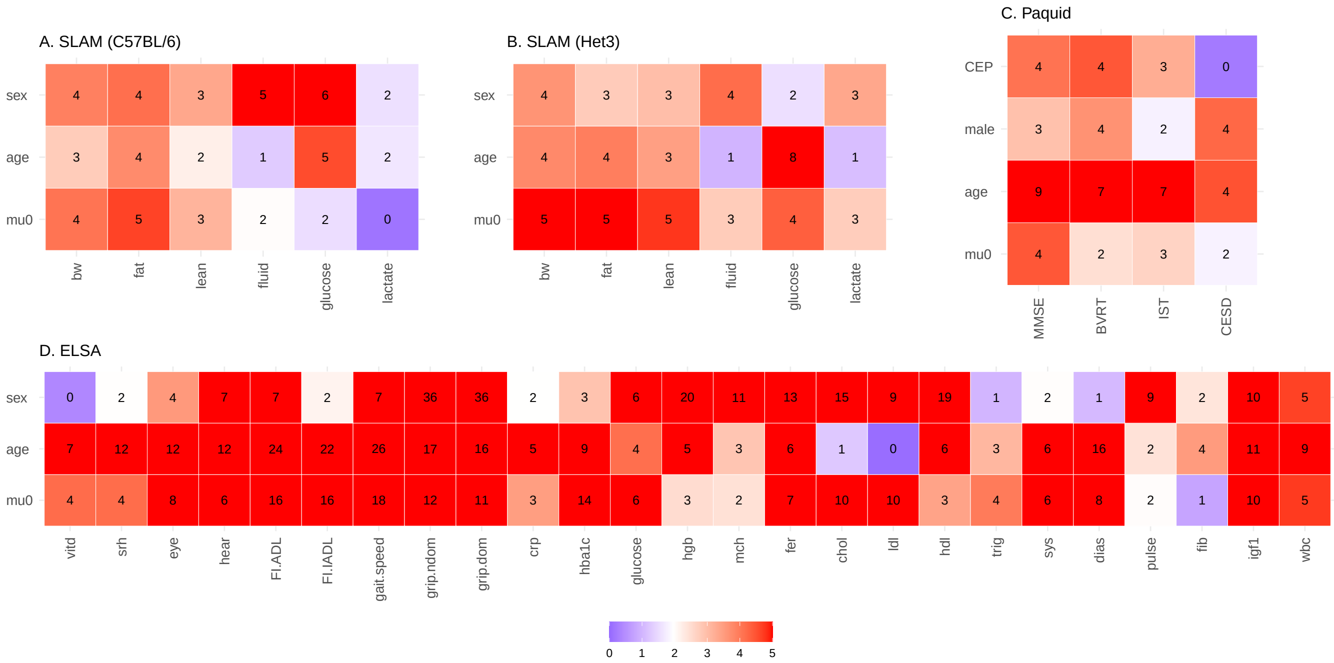

Figure S8. Covariate significance (z-scores). **A.** C57BL/6 mice (SLAM). **B.** Het3 mice (SLAM). **C.** Paquid (human, dementia). **D.** ELSA (human). The equilibrium term, μ, was a linear function of these covariates. Most covariates were significant (red or white). Only the blue tiles were not significant at 95% ($z = 1.96$). Tile number is z-score. Colour scale is truncated at $z = 5$ ($p = 6 \cdot 10^{-7}$). See Figures S11 and S12 for the directions of the covariate effects.

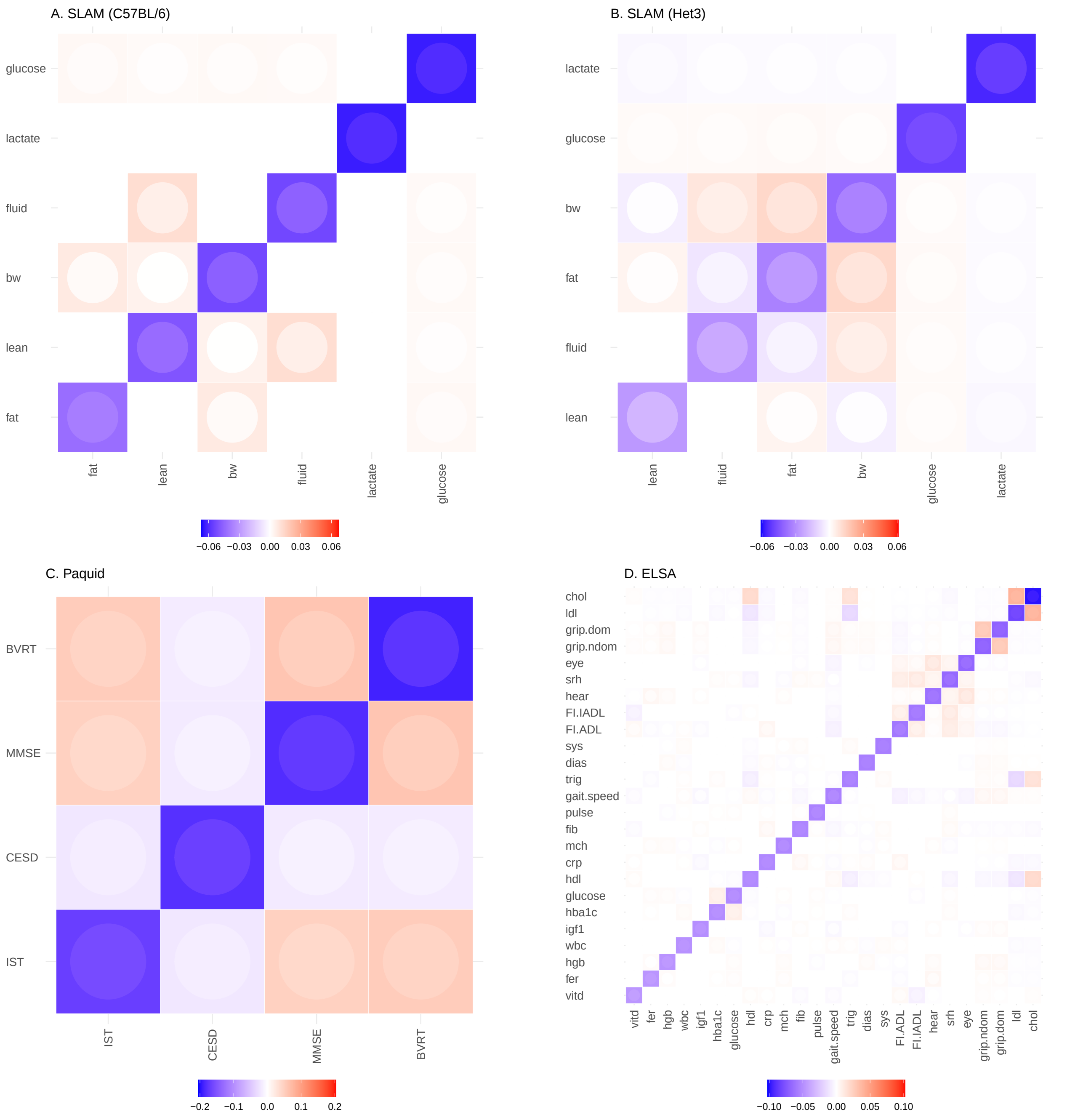

Figure S9. Interaction networks for all datasets. **A.** C57BL/6 mice (SLAM). **B.** Het3 mice (SLAM). **C.** Paquid (human, dementia). **D.** ELSA (human). Tile colour indicates interaction strength (saturation) and direction (colour) of the interaction from the y-axis variable to the x-axis variable. Inner colour indicates the limit of 68% confidence interval (CI) closest to zero (i.e. standard error). Non-significant interactions, at 68%, have been whited-out. Variables are sorted by diagonal strength (increasing rate). The matrices are real and symmetric because the data were diagonalized by an orthogonal matrix (PCA).

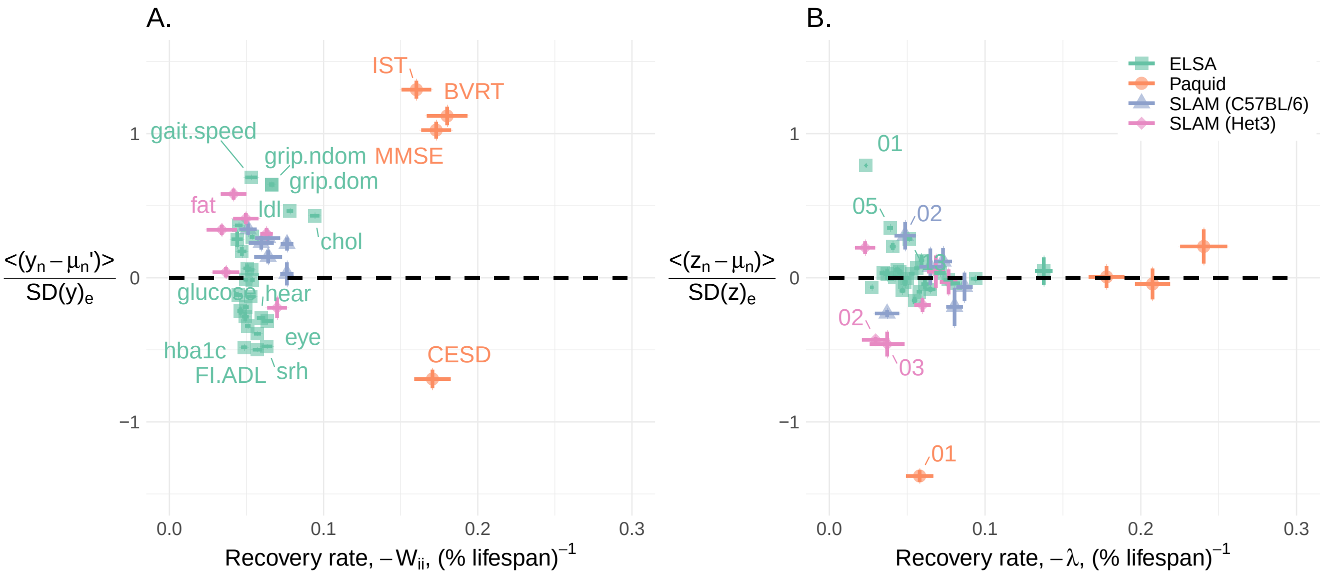

Figure S10. Homeostasis of biomarkers vs natural variables. The dysruption of homeostasis seems to be diffuse across biomarkers whereas it is concentrated into a few natural variables. **A.** Observed biomarkers were typically far from equilibrium (dotted line). **B.** In contrast, most natural variables were close to equilibrium. We inferred that variables close to equilibrium were in homeostasis whereas those far from equilibrium were allostatic. Together these plots suggest that the natural variables were able to condense the effects of allostasis into a few major variables.

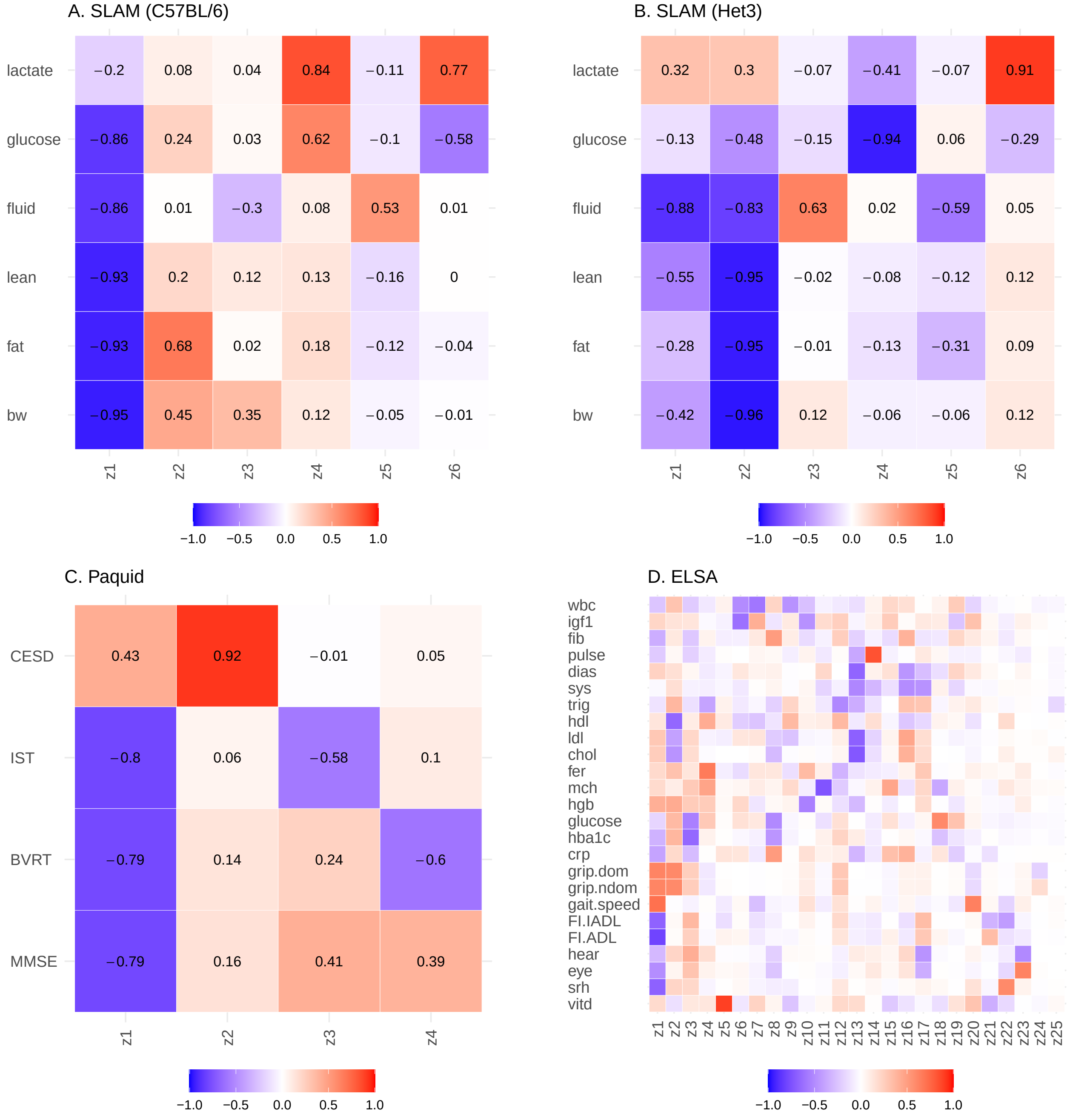

Figure S11. Natural variable correlates — biomarkers (predictors). **A.** C57BL/6 mice (SLAM). **B.** Het3 mice (SLAM). **C.** Paquid (human, dementia). **D.** ELSA (human). This helps to describe what information is in each natural variable, z, and therefore what each natural variable is capable of controlling. The sign of each z is arbitrary due to idiosyncrasies of the eigendecomposition.

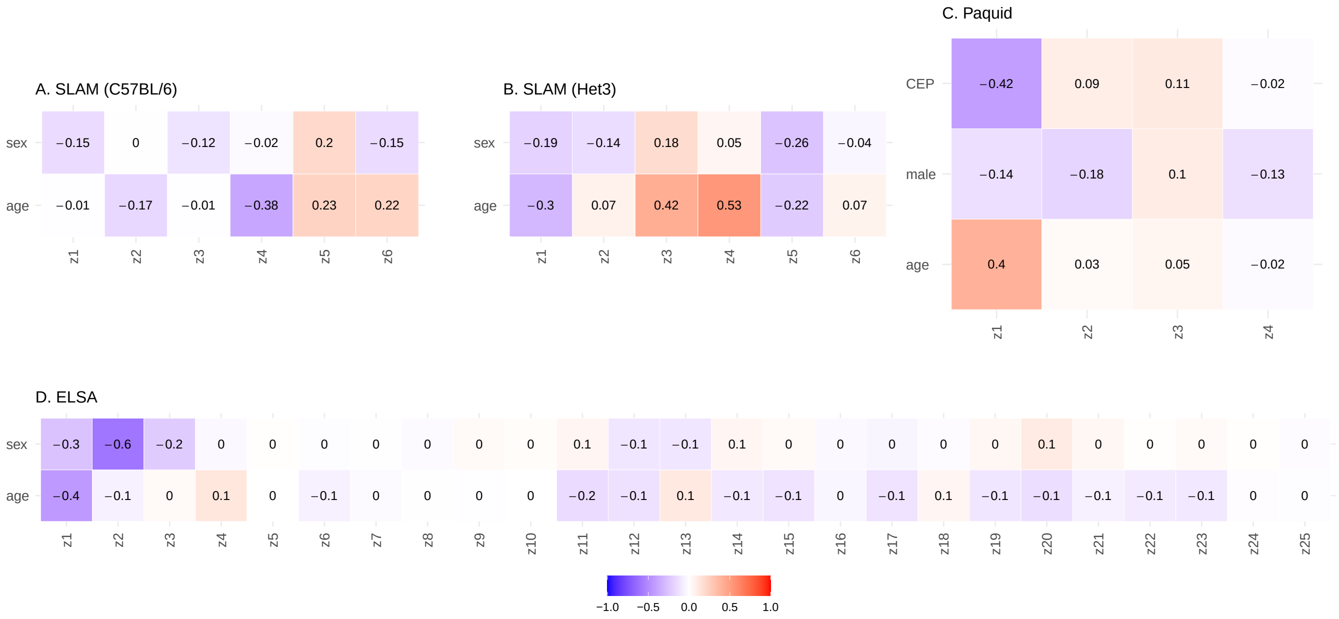

Figure S12. Natural variable correlates — covariates. **A.** C57BL/6 mice (SLAM). **B.** Het3 mice (SLAM). **C.** Paquid (human, dementia). **D.** ELSA (human). This provides further information about what information each natural variable, z, contains. We expect the strongly drifting variables to exhibit correlations with age, though the sign of each z is arbitrary. Male is a binary sex indicator (1: male, 0: female); sex is the converse (0: male, 1: female). CEP is educational attainment level (1: attained primary, 0: did not).

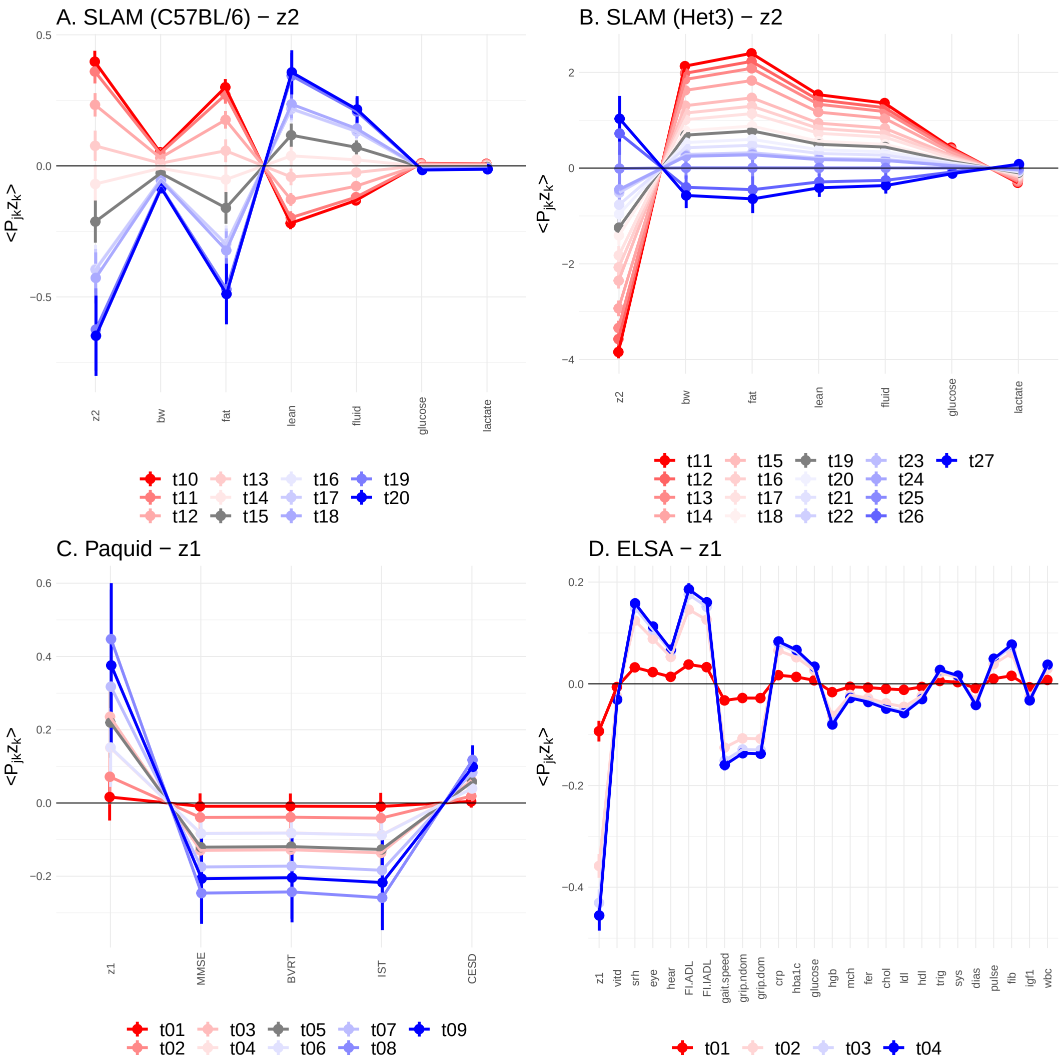

Figure S13. Natural variable drift drives biomarker drift. **A.** C57BL/6 mice (SLAM). **B.** Het3 mice (SLAM). **C.** Paquid (human, dementia). **D.** ELSA (human). We consider the drift of the primary risk natural variables: z_1 for ELSA and Paquid and z_2 for SLAM. We observe a continuous drift in the natural variables. We also plot the drift of the biomarkers which is directly caused by each z via $\boldsymbol{P}$. In this manner, a few natural variables can drive drift across several biomarkers. Since $\boldsymbol{P}$ is orthogonal (length-preserving) the drift of each natural variable must be diluted across biomarkers (at most a single biomarker can drift at the same rate). See also the correlation matrices, Figures S11 and S12. For the SLAM datasets we've included only timepoints where the average age was over 80 weeks.

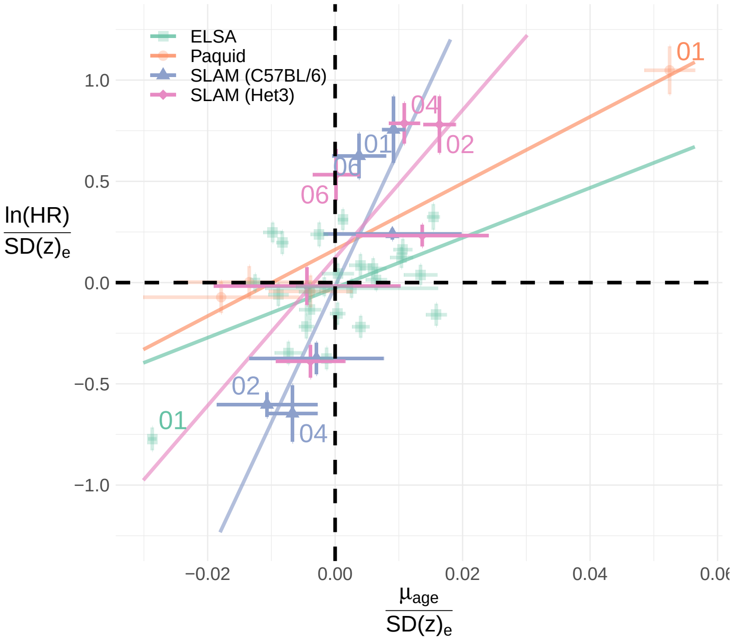

Figure S14. Allostasis drifts towards the risk direction. We fit a Cox model for each natural variable including age and sex as covariates. The Cox coefficient — i.e. log–hazard ratio (HR) per unit increase — correlates with the steady-state drift rate, μ_{age}. The dominant risk direction for each dataset has been labelled by eigenvalue rank (e.g. z_1 is 01). The equilibrium standard deviation provides a native scale for each variable.

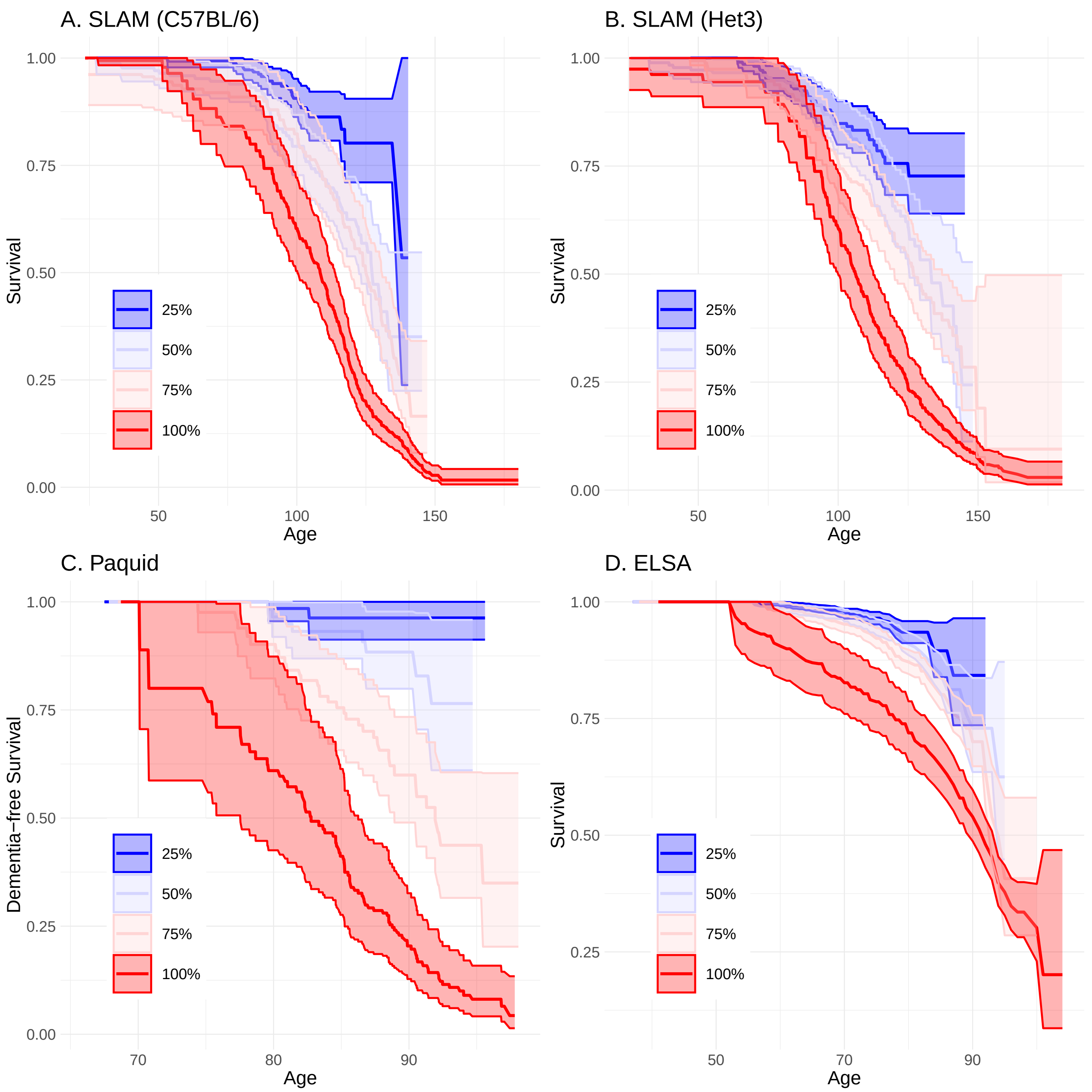

Figure S15. Composite health measure performance. **A.** C57BL/6 mice (SLAM). **B.** Het3 mice (SLAM). **C.** Paquid (human, dementia). **D.** ELSA (human). A simple estimator of health is $\vec{\mu}_{age}^{T}\vec{z}$. This leverages mallostasis to infer individual health. Large separation between quartiles (colours) indicates a strong predictor of adverse outcome. Fill is 95% confidence interval.

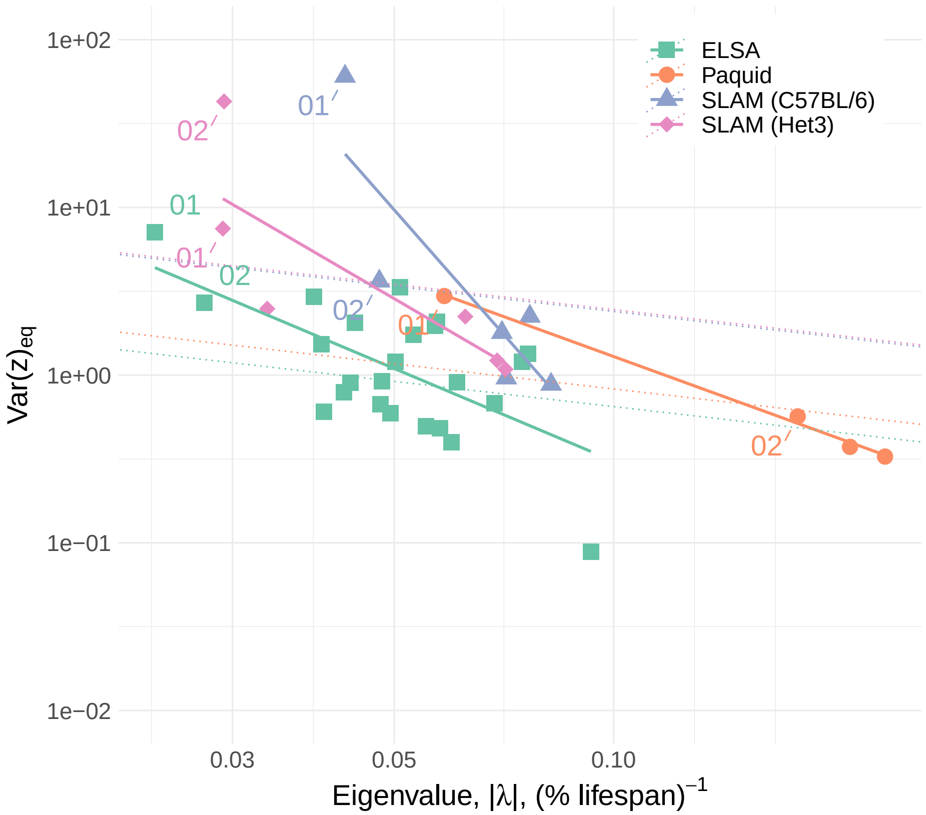

Figure S16. Equilibrium dispersion is primarily determined by eigenvalue strength, $|\lambda|$ equation (S48). Smaller eigenvalues are predicted to have larger equilibrium variances. The range of equilibrium variances spans 3 orders of magnitude. The largest variance will drive the observed variation in biomarkers in the steady-state e.g. rank 1 will become principal component 1 (equation (S49)). Dotted lines illustrate what the equilibrium variance would be if each dimension had the same noise strength, σ^2. The fitted solid lines indicate that the noise makes the smaller eigenvalues even more dominant than expected.

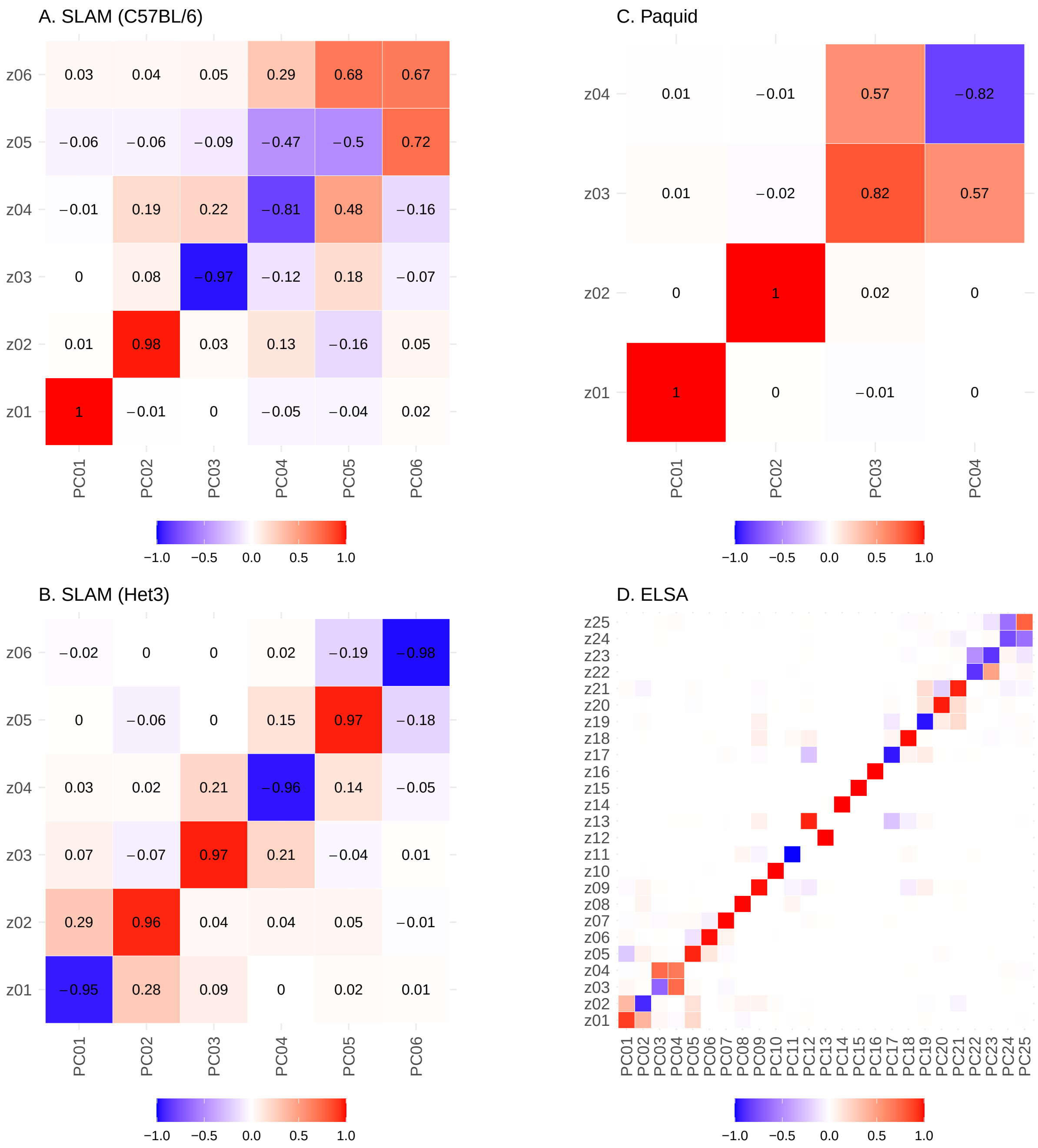

Figure S17. Principal components are very similar to the natural variables. **A.** C57BL/6 mice (SLAM). **B.** Het3 mice (SLAM). **C.** Paquid (human, dementia). **D.** ELSA (human). Shown are the dot products between the principal component rotation and $\boldsymbol{P}$. The dot product assesses similarity between the transformations ranging from 1: identical, 0: orthogonal, and -1: identical with opposing sign. Identical transformations will generate identical natural variables. If the transformations are identical then all values on the diagonal should be ± 1 (sign is arbitrary[12]). We see that the dot products are often close to ± 1, indicating that the transformations are very close, although they do not perfectly coincide.

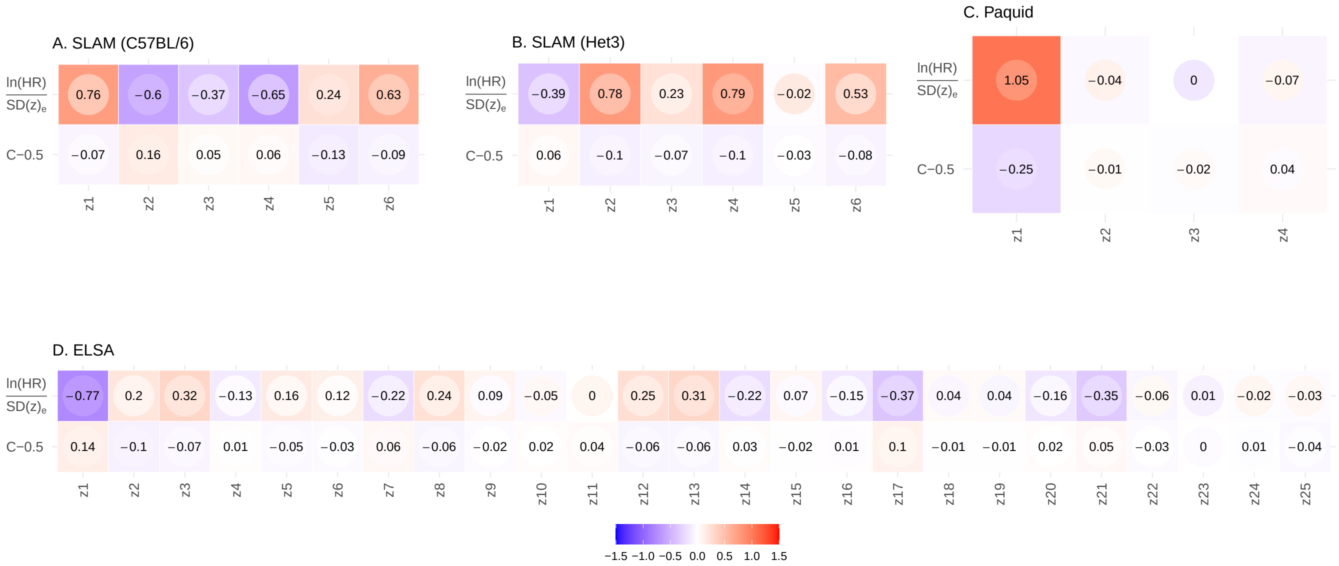

Figure S18. Survival summary. **A.** C57BL/6 mice (SLAM). **B.** Het3 mice (SLAM). **C.** Paquid (human, dementia). **D.** ELSA (human). For each dataset, the top row corresponds to the Cox coefficient standardized by the equilibrium dispersion ($\ln(HR)/SD(z)_e$) while the bottom is the C-index centered to 0 ($C-0.5$). A Cox coefficient greater than 0 indicates that higher values are at increase risk and vice versa. A centered C-index greater than 0 indicates that higher values are at reduced risk and vice versa (opposite of the Cox coefficient). The Cox model is conditioned on age and sex (the same as Figure S14); the C-index is unconditioned. We see that in humans, the first dimension is the dominant determinant in risk of death (ELSA) or dementia (Paquid). It is less clear in mice, where allostatic drift is a better way to identify important survival dimensions (Figure S14 or Figure 4A). Inner colour indicates the limit of 95% confidence interval (CI) closest to zero (non-significant are red on blue or blue on red).

References

1. Edemekong, P. F., Bomgaars, D. L., Sukumaran, S. & Levy, S. B. *Activities of Daily Living* (StatPearls Publishing, Treasure Island (FL), 2021).
2. Searle, S. D., Mitnitski, A., Gahbauer, E. A., Gill, T. M. & Rockwood, K. A standard procedure for creating a frailty index. *BMC Geriatr.* **8**, 24, DOI: 10.1186/1471-2318-8-24 (2008).
3. Palliyaguru, D. L. *et al.* Fasting blood glucose as a predictor of mortality: Lost in translation. *Cell Metab.* **33**, 2189–2200.e3, DOI: 10.1016/j.cmet.2021.08.013 (2021).
4. Proust-Lima, C., Philipps, V. & Liquet, B. Estimation of extended mixed models using latent classes and latent processes: The R package lcmm. *J. Stat. Softw.* **78**, 1–56, DOI: 10.18637/jss.v078.i02 (2017).
5. Sterne, J. A. C. *et al.* Multiple imputation for missing data in epidemiological and clinical research: potential and pitfalls. *BMJ* **338**, b2393, DOI: 10.1136/bmj.b2393 (2009).
6. van Buuren, S. & Groothuis-Oudshoorn, K. mice: Multivariate imputation by chained equations in R. *J. Stat. Softw.* **45**, 1–68 (2010).
7. Petersen, Kaare, Brandt and Pedersen, Michael, Syskind. The matrix cookbook. Online https://www.math.uwaterloo.ca/~hwolkowi/matrixcookbook.pdf (2012).
8. Wickham, H. ggplot2: Elegant graphics for data analysis (2016).
9. Hardy, S. E., Allore, H. & Studenski, S. A. Missing data: a special challenge in aging research. *J. Am. Geriatr. Soc.* **57**, 722–729, DOI: 10.1111/j.1532-5415.2008.02168.x (2009).
10. Bender, R., Augustin, T. & Blettner, M. Generating survival times to simulate cox proportional hazards models. *Stat. Med.* **24**, 1713–1723, DOI: 10.1002/sim.2059 (2005).
11. Hastie, T., Tibshirani, R. & Friedman, J. *The elements of statistical learning: data mining, inference, and prediction*, vol. 2nd (Springer, 2017).
12. Pridham, G., Rockwood, K. & Rutenberg, A. Efficient representations of binarized health deficit data: the frailty index and beyond. *Geroscience* **45**, 1687–1711, DOI: 10.1007/s11357-022-00723-z (2023).
13. Xiaogang, X. A., Yan. *Linear Regression Analysis: Theory And Computing* (World Scientific, 2009).
14. Held, L. & Sabanés Bové, D. *Applied Statistical Inference: Likelihood and Bayes* (Springer, Berlin, Heidelberg, 2014).
15. Henderson, D. & Plaschko, P. *Stochastic Differential Equations In Science And Engineering (with cd-rom)* (World Scientific, 2006).
16. Byron, F. W. & Fuller, R. W. *Mathematics of Classical and Quantum Physics* (Dover, 1992).
17. Sehl, M. E. & Yates, F. E. Kinetics of human aging: I. rates of senescence between ages 30 and 70 years in healthy people. *J. Gerontol. A Biol. Sci. Med. Sci.* **56**, B198–208, DOI: 10.1093/gerona/56.5.b198 (2001).
18. Mitnitski, A. & Rockwood, K. Aging as a process of deficit accumulation: its utility and origin. *Interdiscip. Top. Gerontol.* **40**, 85–98, DOI: 10.1159/000364933 (2015).
19. Ledder, G. *Mathematics for the Life Sciences* (Springer New York, 2013).
20. Yashin, A. I. *et al.* Stochastic model for analysis of longitudinal data on aging and mortality. *Math. Biosci.* **208**, 538–551, DOI: 10.1016/j.mbs.2006.11.006 (2007).
21. Farrell, S., Mitnitski, A., Rockwood, K. & Rutenberg, A. D. Interpretable machine learning for high-dimensional trajectories of aging health. *PLoS Comput. Biol.* **18**, e1009746, DOI: 10.1371/journal.pcbi.1009746 (2022).
22. Mitchell, S. J., Scheibye-Knudsen, M., Longo, D. L. & de Cabo, R. Animal models of aging research: implications for human aging and age-related diseases. *Annu. Rev Anim Biosci* **3**, 283–303, DOI: 10.1146/annurev-animal-022114-110829 (2015).
